# *Agathis* vs*. Hymenaea*—trapping biases to interpret arthropod assemblages in ambers

**DOI:** 10.1186/s12915-025-02453-y

**Published:** 2025-11-07

**Authors:** Mónica M. Solórzano-Kraemer, Antonio Monleón-Getino, Enrique Peñalver, Atahualpa S. Kraemer, Mélanie C. M. Herbert, David Peris, Antonio Arillo, Vincent Perrichot, Eduardo Barrón, Romain Garrouste, Maria Paulsen, Xavier Delclòs

**Affiliations:** 1 Senckenberg Research Institute, Frankfurt Am Main, 60325 Germany; 2 GRBIO3, Section of Statistics, Department of Genetics, Microbiology, and Statistics, Faculty of Biology, University of Barcelona, Barcelona, 08028 Spain; 3 Instituto Geológico y Minero de España, IGME-CSIC, Valencia, 46004 Spain; 4 Departamento de Física, Facultad de Ciencias, Universidad Nacional Autónoma de México, Ciudad Universitaria, Mexico City, 04510 Mexico; 5 Institut Botànic de Barcelona (CSIC-CMCNB), Barcelona, 08038 Spain; 6 Departamento de Biodiversidad, Ecología y Evolución, Facultad de Biología, Universidad Complutense, Madrid, 28040 Spain; 7 Géosciences Rennes (UMR 6118), Univ Rennes, CNRS, Rennes, 35000 France; 8 Instituto Geológico y Minero de España, IGME-CSIC, Madrid, 28003 Spain; 9 Institut de Systématique, Evolution Biodiversité (ISYEB), UMR 7205—CNRS, MNHN—Sorbonne Univ.-EPHE-Univ. des Antilles, Muséum National d’Histoire Naturelle, CP50 Entomologie, Paris, 75005 France; 10 School of Earth, Atmosphere, and Environment, Monash University, Clayton, VIC 3800 Australia; 11 Departament of Earth and Ocean Dynamics, Faculty of Earth Sciences, University of Barcelona, Barcelona, 08028 Spain; 12 Biodiversity Research Institute (IRBio), University of Barcelona, Barcelona, 08028 Spain

**Keywords:** Amber, Actuotaphonomic studies, Cretaceous, Miocene, Biocoenosis, Taphonomy, Copal, Defaunation resin

## Abstract

**Background:**

The genera *Agathis* (Coniferales: Araucariaceae) and *Hymenaea* (Fabales: Fabaceae) contain resin-producing tree species that are crucial for actuotaphonomic studies. While certain Cretaceous ambers likely originated from *Agathis* or *Agathis*-like trees, *Hymenaea* is the primary source of many Miocene ambers. Field studies were conducted in New Caledonia and Madagascar to collect Defaunation resin (resin produced after 1760 AD (Anno Domini)). Arthropods were collected with yellow sticky and Malaise traps in New Caledonia, Madagascar, and Mexico. Cretaceous and Miocene ambers, copals (2.58 Ma to 1760 AD), and Defaunation resins from various regions were analysed to compare arthropod trapping patterns.

**Results:**

Actuotaphonomic results show lower number of arthropods trapped in *Agathis* Defaunation resin, with a non-uniform distribution, compared to the abundant and uniformly distributed arthropods trapped in *Hymenaea* Defaunation resin. The lower number of arthropod inclusions in the trunk resin of the *Agathis* trees is attributed to the rapid polymerisation of that resin. Under the same experimental conditions, the arthropods in *Agathis* Defaunation resin plot far from the arthropods collected in the yellow sticky and Malaise traps, while the arthropods in *Hymenaea* Defaunation resin plot close to the arthropods in the yellow sticky traps.

**Conclusions:**

These findings confirm different resin trapping patterns between *Agathis* and *Hymenaea*, with significant implications for interpreting the amber record. The fauna trapped by *Hymenaea* resin closely resembles the arthropod biocoenosis that live in and around the trunks, indicating autochthony and close relationship with the forest ecosystem, unlike *Agathis* resin. These results improve our understanding of arthropod trapping biases in resin and lead us to reconsider previously proposed interpretations of Cretaceous forest biocoenoses.

**Supplementary Information:**

The online version contains supplementary material available at 10.1186/s12915-025-02453-y.

## Background

Amber is a fossil plant resin that sometimes preserves inorganic inclusions such as water or soil particles and a high diversity of bioinclusions, such as phloem sap, plant remains, microorganisms, invertebrates (with arthropods being the most abundant), and vertebrate remains, providing a unique window into biocoenoses in ancient forested ecosystems [1–4]. In the Cretaceous period, conifers were globally the primary contributors to sedimentary deposits of resin (today amber) [5]. Several families have been proposed as the producers of large quantities of resin at that time, namely Araucariaceae, Cupressaceae, Pinaceae, Podocarpaceae, and the extinct Cheirolepidiaceae [6–9]. Moving to the Eocene epoch, amber deposits were derived from conifers and angiosperms. For example, Eocene Baltic amber originated from a conifer tree of the family Pinaceae and/or Cupressales: Sciadopityaceae [10, 11], while Cambay (India) amber was linked to the angiospermous Dipterocarpaceae [12]. Conversely, the majority of Miocene ambers, copals, and Defaunation resins (*sensu* Solórzano-Kraemer et al. [13]) were derived from angiosperms, predominantly of the families Fabaceae and Dipterocarpaceae [13, 14].

The Cretaceous, Paleogene, and Neogene periods are the most prolific in terms of described amber deposits [5, 13]. However, a difference has been noted in unbiased collections between the amber that originated from conifers and that from angiosperms. Of the approximately 500 Barremian amber localities known in Lebanon, only 38 contain arthropod inclusions [15, 16]. Similarly, of the more than 140 Albian amber localities in Spain, only 11 contain arthropod inclusions [17, 18]; of these, four are poor and contain only one or two arthropod inclusions. Another example is the Albian–Cenomanian French amber, with 60 reported localities, of which 14 contain arthropod inclusions, but only one, Archingeay-Les Nouillers, was reported as highly fossiliferous [19–23]. Cretaceous amber from Southern America seems to exhibit a similar circumstance, as arthropod inclusions have remained elusive despite many years of intensive searching [24–26]. Only 21 terrestrial arthropods in Ecuadorian Albian amber are known until now from South America [27]. It is important to consider that many Cretaceous amber deposits mentioned in the literature contain only an anecdotal amber record. Deposits with significant amber quantities are scarce [5], and researchers primarily focus on amber-rich deposits when searching for arthropod inclusions.

On the contrary, Cenozoic amber localities (from the Paleogene and Neogene) appear less abundant than in the Cretaceous, but nearly all contain arthropod inclusions [13, 28]. However, the inclusion density, i.e. the number of specimens per volume or mass of inspected amber, has never been studied before. These observations raise question on whether the difference in arthropod inclusions is due to a different distribution of fauna in the ecosystem or a different trapping bias of the resins.

Resin has been defined as an entomological trap that works similarly to commercial yellow sticky traps [29, 30]. The fauna living in and around the resin-producing trees of *Hymenaea* Linnaeus, 1753 has been described as having a greater chance of being trapped and preserved in the resin (the so-called thanatocoenosis or death assemblage), than the fauna living in surrounding areas of the forest [30]. Some resins often contain inclusions that are closely related to the habitats provided by resiniferous trees. Diptera is an order that contains a high number of inclusions in amber. Most of these insects are fungivores, carnivorous, necrophagous, or hematophagous, which explains why they are commonly found as amber inclusions [30]. *Agathis* Salisbury, 1807 (Araucariaceae) and *Hymenaea* (Fabaceae) genera contain resin-producing tree species serving as pivotal models for actuotaphonomic investigations in this respect [29–32]. The genus *Agathis* is primarily distributed today in the Southern Hemisphere, growing in tropical environments across the Malay Archipelago (Malesia), the islands of the South-West Pacific (Melanesia), northern New Zealand, and South Queensland in Australia [33]. *Agathis* or *Agathis*-like trees have been postulated to be the source of certain Cretaceous and Cenozoic ambers from Lebanon (Barremian), Spain (Albian), France (Albian–Cenomanian), Myanmar (Cenomanian), Australia (Turonian–Campanian), and New Zealand (Oligocene and Miocene), while there is certainty that *Agathis* was or is involved in the origin of the copals and Defaunation resins from New Zealand and New Caledonia [3, 13, 34, 35]. *Hymenaea* is a tropical and subtropical resin-producing tree originating in East Africa during the early Miocene, mainly distributed throughout Central and South America, and East Africa (including Kenya, Tanzania, Mozambique, Comoros, Madagascar, Mauritius, and Reunion Island) [36–39]. The genus *Hymenaea* has been identified as the source of the ambers from Ethiopia, Mexico, and Dominican Republic (all Miocene in age), Pleistocene and Holocene copals, and Defaunation resins from East Africa, and Central and South America [13, 39, 40].

Understanding why many amber deposits are poor or devoid in arthropod inclusions, and why this holds true for Cretaceous deposits more than for Paleogene and Neogene ones, would contribute to a better interpretation of oryctocoenoses (fossil assemblages) based on amber records. We have thus carried out a comparative actuotaphonomic study based on *Agathis* trees from New Caledonia and *Hymenaea* trees from Madagascar. We followed the same experimental protocol as in a previous study based on *Hymenaea* from Madagascar [30] in order to compare the results. Our aim was to quantify, using statistical analysis, the differences in the intensity of arthropod trapping between the resin exuded by two different resin-producing trees (“gymnosperms” exclusively conifers vs. angiosperms) in their natural environments, with a view to applying the conclusions to the reconstruction of deep-time environments based on fossil assemblages preserved in amber.

A Spanish translation of the abstract is provided in Additional file 1.

## Results

### Trapping patterns of arthropods in ambers, copals, and Defaunation resins from *Agathis*, *Agathis*-like, and *Hymenaea* trees

**Fig. 1:**
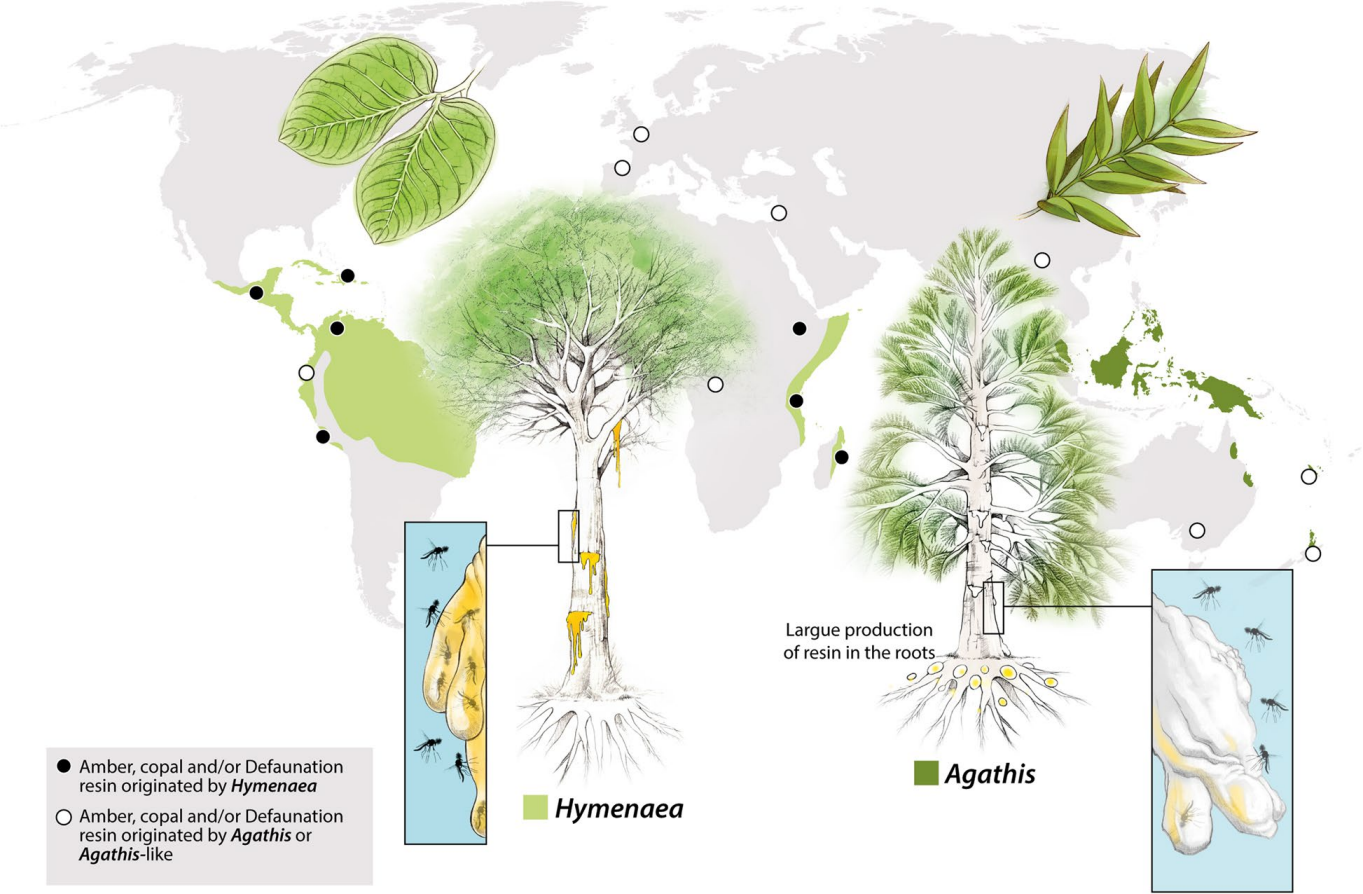
Schematic summary of the main differences between the two primary resin-producing genera, *Agathis* and *Hymenaea*. Dark green shading indicates the current geographic distribution of *Agathis*, while light green shading shows that of *Hymenaea*. The relative whiteness of the resin reflects the speed of hardness in *Agathis*. Amber, copal, or Defaunation resins from most of the localities represented here are included in this study (see Table 1 and Additional file 2). Large lumps of resin are produced only in the roots of the *Agathis*. The schematic also illustrates the effectiveness of each resin type as an entomological trap for arthropods

**Fig. 2:**
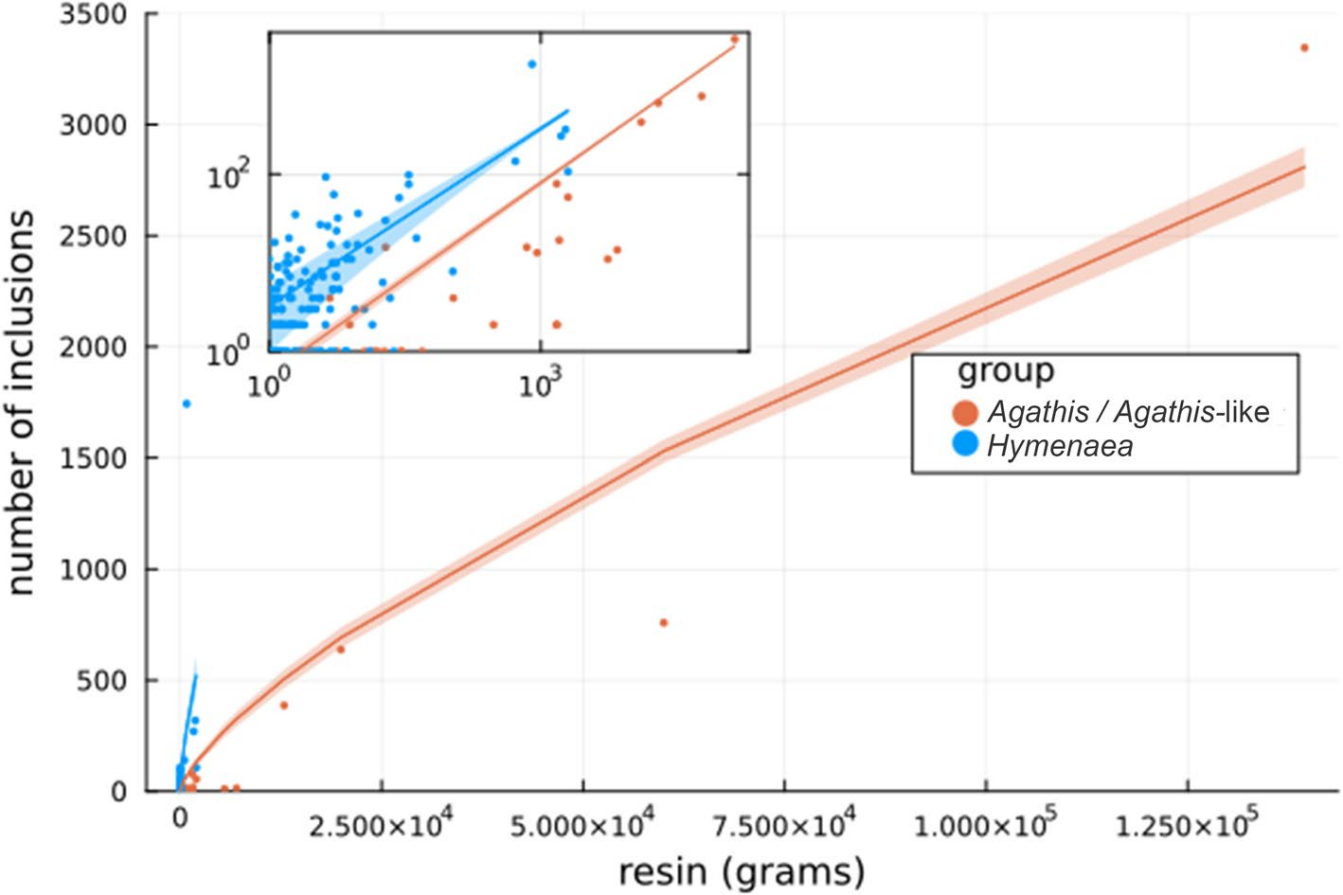
Scatter plot showing the number of arthropod inclusions as a function of resin mass (in grams) for *Agathis* and *Agathis*-like samples (red–orange) and *Hymenaea* samples (blue). The inset displays the same data in log–log scale. Continuous lines represent fitted power-law functions, with exponents constrained between ⅔ and 1. Shaded bands around the fits indicate the standard error of the estimates. The dataset used to generate this figure is available in Additional file 4. Spanish, French, Australian, New Zealand, and New Caledonian resin exudates were produced by conifers; Dominican, Mexican, and Malagasy resin exudates were produced by angiosperms

In the Defaunation resin obtained from *Agathis* in New Caledonia (Additional file 2: Fig. S1), a total of 48 arthropod inclusions were found in 2618.7 g of resin (Table 1), mostly Acari, Pseudoscorpiones, Collembola, and non-flying Hymenoptera, namely Formicidae, but also a Thysanoptera and a Diptera remain. This scarce number of arthropods in *Agathis* Defaunation resin was unexpected, particularly considering the significant number of arthropod inclusions (~ 4000) found in 2300 g of Malagasy Defaunation resin from *Hymenaea* (Table 1, see Additional file 3 for a detailed list of arthropod inclusions). We observed a visible divergence in arthropod trapping efficiency (Fig. 1). Here, efficiency refers to two things: First, it refers to the increased trapping of arthropods and other organisms by both ancient and modern resins. Second, it refers to the recording of a more complete (varied) assemblage that better reflects the communities that inhabited these ancient and modern forests. We also observed a visible divergence in arthropod trapping patterns, represented in Fig. 2, between *Agathis* and *Agathis*-like, and *Hymenaea* samples (including ambers and copal from both types of trees). When comparing arthropod counts with resin mass, two different trends emerge: With the increase in resin mass, the pattern of *Agathis* and *Agathis*-like samples show a slow, near-flat increase. Meanwhile, *Hymenaea* samples show a sharp increase, indicating markedly higher trapping efficiency.

**Table 1:** Number of arthropod inclusions per gram in amber, copal, or Defaunation resin that are reported in the literature and own data

Locality/area of collection	Age of resin	Resiniferous tree	Weight/pieces	N. of arthropod inclusions	Citation
Mananjary region (between Nosy Varika and Ambahy), Madagascar	Resin collected in 2013	*Hymenaea verrucosa* (Caesalpiniaceae)	800.5 g	1743	Solórzano-Kraemer et al. [30]
Sacaramy (close to Antsiranana, Diego Suarez), Madagascar	Resin collected in 2015	*Hymenaea verrucosa* (Caesalpiniaceae)	1500 g	2141	Own data
Col de Yaté South Province (Gramseat South), New Caledonia	Resin collected in 2016	*Agathis ovata* (Araucariaceae)	1627.1 g	40	Own data
Bon Secours (close to Rivière Bleue Provincial Park, South Province, Gramseat South), New Caledonia	Resin collected in 2016	*Agathis lanceolata* (Araucariaceae)	991.6 g	8	Own data
Copal/Defaunation resin from Cotuí, Dominican Republic	Unknown	*Hymenaea courbaril* (Caesalpiniaceae)	1875.2 g	319	Own data
Amber from Totolapa, Chiapas, Mexico	Early Miocene	†*Hymenaea mexicana* Poinar and Brown, 2002 (Caesalpiniaceae)	2000 g	107	Solórzano-Kraemer et al. [29]
Amber from El Valle 7 Cañadas, Dominican Republic	Early Miocene	†*Hymenaea protera* Poinar, 1991 (Caesalpiniaceae)	1678.8 g	270	Own data
Amber from San Rafael, Dominican Republic	Early Miocene	†*Hymenaea protera* (Caesalpiniaceae)	523.5 g	140	Own data
Amber from Southland region of the South Island, and Otago, New Zealand	Late Oligocene and early Miocene	*Agathis* sp. (Araucariaceae)	The exact amount is unknown, estimation: 1000 to 1500 g	78	Schmidt et al. [41] (Alexander Schmidt, pers. comm., February 2023)
Amber from Anglesea Coal Measures (ACM), Australia	Early Eocene	*Agathis sp.	ca. 2000 g	47	Stilwell et al. [35], own data 2025
Amber from Macquarie Harbour Formation (MHF), Australia	Early Eocene	*Agathis sp.	ca. 1500 g	2	Stilwell et al. [35], own data 2025
Amber from Fourtou, France	Middle Cenomanian	†*Agathoxylon* sp. (Araucariaceae) or †Cheirolepidiaceae	ca. 2000 g	40	Girard et al. [42], own data 2025
Amber from Fouras/Bois Vert, France	Early Cenomanian	†*Agathoxylon gardoniense* (Araucariaceae) and †Cheirolepidiaceae	ca. 3000 g	113	Néraudeau et al. [43], Perrichot et al. [21], own data 2025
Amber from Ile d’Aix, France	Early Cenomanian	†*Agathoxylon gardoniense* (Araucariaceae) and †Cheirolepidiaceae	ca. 500 g	6	Néraudeau et al. [44], own data 2025
Amber from La Buzinie, France	Early Cenomanian	†*Agathoxylon gardoniense* (Araucariaceae) and †Cheirolepidiaceae	ca. 6000 g	149	Perrichot et al. [21], own data 2025
Amber from Salignac, France	Early Cenomanian	Araucariaceae	ca. 400 g	27	Perrichot et al. [21], own data 2025
Amber from Archingeay-Les Nouillers, France	Late Albian	†*Agathoxylon gardoniense* (Araucariaceae) and †Cheirolepidiaceae	35,000 to 40,000 g	1330	Perrichot et al. [21], own data 2025
Amber from Les Renardières, France	Late Albian	†*Agathoxylon gardoniense* (Araucariaceae) and †Cheirolepidiaceae	300 g	4	Perrichot et al. [21], own data 2025
Amber from Cadeuil, France	Late Albian	†*Agathoxylon gardoniense* (Araucariaceae) and †Cheirolepidiaceae	ca. 8000 g	98	Néraudeau et al. [45], own data 2025
Amber from Peñacerrada, Álava, Spain	Late Albian (Early Cretaceous)	*Agathis*-like (Araucariaceae) (Chaler and Grimalt [46])	139,500 g	3346	Own data
Amber from San Just, Teruel, Spain	Late Albian (Early Cretaceous)	*Agathis*-like (Araucariaceae)	12,900 g	387	Own data
Amber from La Hoya, Castellón, Spain	Early Cenomanian (Early Cretaceous)	*Agathis*-like (Araucariaceae)	5500 g	11	Own data
Amber from El Soplao, Cantabria, Spain	Middle Albian (Early Cretaceous)	*Frenelopsis* sp. (†Cheirolepidiaceae) and other possible Cupressaceae (Menor-Salván et al. [47])	19,937 g	1600	Own data
Amber from Arroyo de la Pascueta, Teruel, Spain	Late Albian (Early Cretaceous)	*Agathis*-like (Araucariaceae)	7000 g	14	Own data
Amber from La Rodada, La Manjoya, Spain	Late Albian (Early Cretaceous)	*Agathis*-like (Araucariaceae)	500 g	2	Peñalver et al. [48]
Amber from Ariño, Teruel, Spain	Early Albian (Early Cretaceous)	*Agathis*-like (Araucariaceae) (Álvarez-Parra et al. [49])	1128 g	100	Own data
Amber from Doumanga, Congo	Middle Aptian	†*Agathoxylon* sp. (Araucariaceae) or †Cheirolepidiaceae	2550 g	47	Bouju and Perrichot [50], own data 2025

The amber, copal, and Defaunation resin (collected directly from the trees) from the different areas were not pre-selected in terms of containing or not containing arthropod inclusions. The table includes collections that are unbiased in terms of arthropods per gram; unbiased collections only in terms of no pre-selection of arthropod species are not included here.*Stilwell et al. [35] do not specify a source for this amber, but its age and provenance suggest *Agathis* sp

**Fig. 3:**
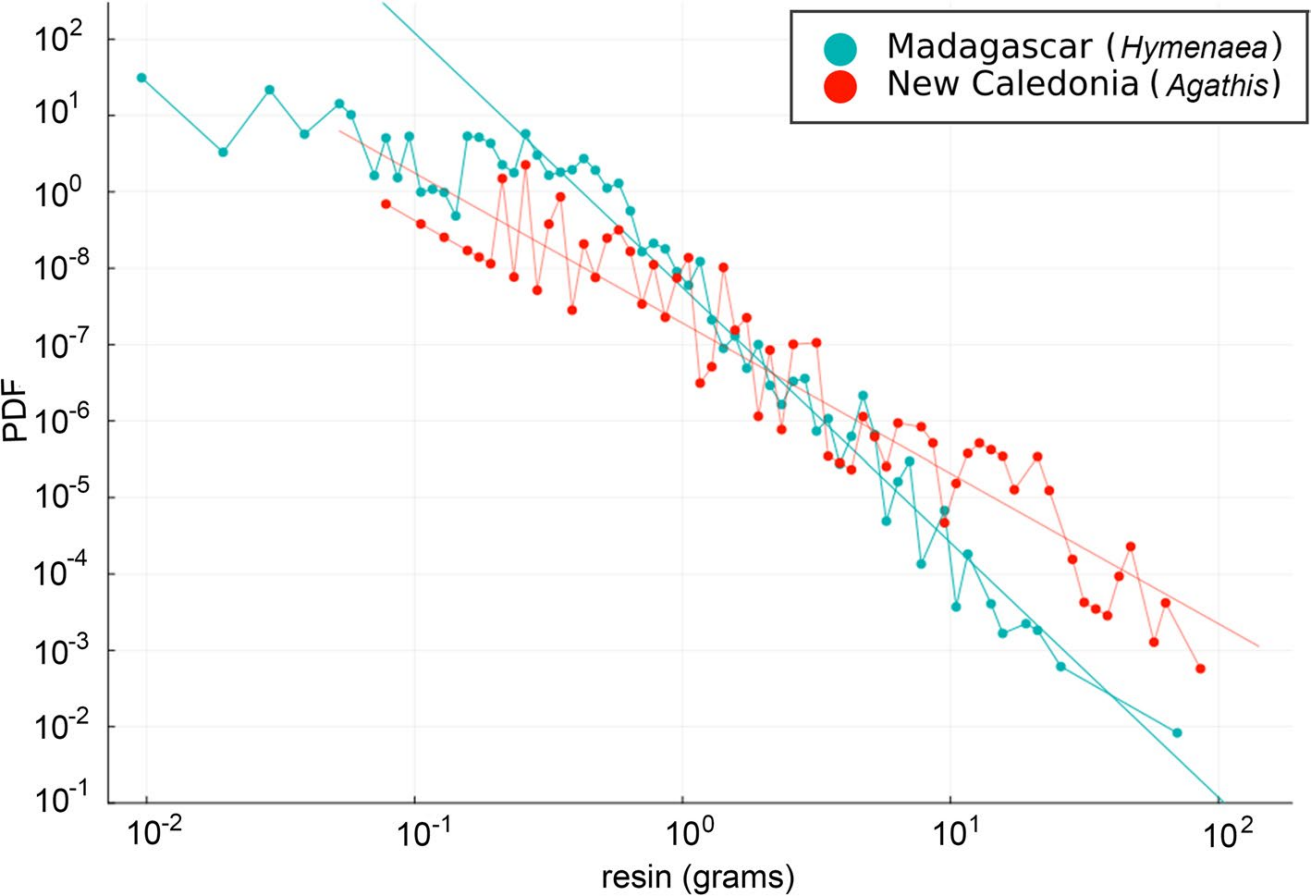
Log–log plot of the probability distribution function (PDF). PDF of finding a piece of Defaunation resin without arthropod inclusions given that the piece has a mass *m* [resin (grams)] for Defaunation resins from *Agathis* (New Caledonia) and *Hymenaea* (Madagascar) trees. Both distributions follow a power law, with exponents of *b* = − 1.97 for *Agathis* Defaunation resin and *b* = − 3.34 for *Hymenaea* Defaunation resin. The exponent *b* indicates the uniformity of the distribution, with more negative values approaching a uniform system (see Additional file 2 for further explanation). Note that in the case of the *Hymenaea* Defaunation resin, with collections in which the smaller pieces only weight 0.01 g, a change in the behaviour of the curve appears at around 0.5 g. This change in trend is likely due to human bias during collection. For this reason, we did not use the data corresponding to pieces smaller than 0.5 g to obtain the exponent *b*

The different arthropod trapping patterns observed in the *Hymenaea* and *Agathis* Defaunation resins are revealed not only by the number of arthropod inclusions per mass of resin (Fig. 2), but also by the probability that a collected Defaunation resin piece/lump with a certain mass does not have any inclusions. The thanatocoenosis (death assemblages) varies greatly between that of *Agathis* Defaunation resin and that of *Hymenaea* Defaunation resin, which will considerably influence the interpretation of the resulting oryctocoenosis. The probability density function (PDF) of resin pieces lacking arthropod inclusions was analysed in relation to mass for *Hymenaea* (Madagascar) and *Agathis* (New Caledonia) Defaunation resins (Fig. 3). The distributions for both types of resin followed a power-law trend, with exponents of *b* = − 1.97 and *b* = − 3.34 for *Agathis* and *Hymenaea*, respectively. The exponent *b* is a measure of distribution uniformity: values closer to zero indicate greater variability, while more negative values suggest a system approaching uniformity (an explanation of PDF and the degree of uniformity is presented in Additional file 2). For *Hymenaea*, the probability decreases rapidly as the mass increases; for *Agathis*, however, the decrease is gradual. This reflects the fact that large *Agathis* pieces lack arthropod inclusions. The rate at which the probability decreases reflects the distribution of arthropod inclusions. A uniform distribution leads to an exponential decrease corresponding to an infinite exponent *b*, whereas a distribution in which all inclusions are concentrated in a single location would have an exponent of *b* = 0. Thus, the value of *b* serves as an indicator of the uniformity of the distribution of arthropod inclusions in amber. If the only difference were a lower arthropod density around *Agathis* and both resins trapped them equally, the lines would have the same shape but offset from each other and they would appear parallel. Therefore, the different values of *b* for each collection of pieces of Defaunation resin show that *Hymenaea* resin traps arthropods more efficiently and more uniformly than *Agathis* resin (see also Additional file 2: Fig. S1 and extended material and methods in Additional file 2). In other words, the characteristics of the thanatocoenosis are very different for each type of resin under investigation here.

### Arthropod trapping patterns including yellow sticky and Malaise traps

**Fig. 4:**
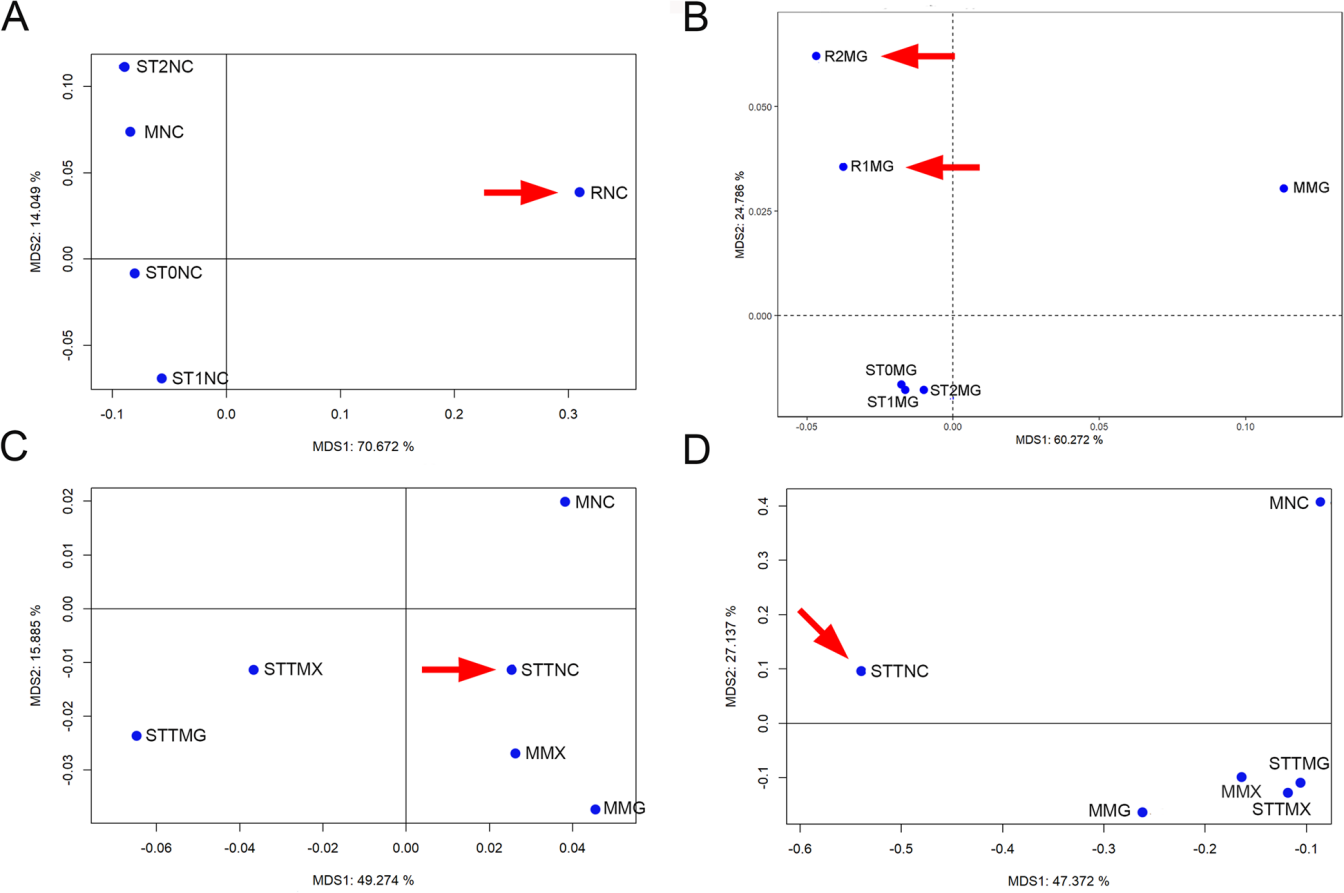
Multidimensional scaling (MDS) using Bhattacharyya distance for arthropod orders (**A**–**C**) and Diptera families (**D**) trapped on the trunk and close to *Agathis* and *Hymenaea* trees. **A** By the yellow sticky and Malaise traps and by Defaunation resin in an *Agathis* forests in New Caledonia. **B** By the yellow sticky and Malaise traps and by Defaunation resin in an *Hymenaea* forests in Madagascar. **C** By the yellow sticky and Malaise traps in an *Agathis* forest in New Caledonia and in an *Hymenaea* forests in Madagascar and Mexico. **D** By yellow sticky and Malaise traps in an *Agathis* forest in New Caledonia and in an *Hymenaea* forests in Madagascar and Mexico. The MDS plot shows the proportion of variance explained by each component of the Bhattacharyya distance scaling for each axis. Red arrows: In **A**, arthropods trapped by the *Agathis* Defaunation resin are far from the Malaise traps and yellow sticky traps. In **B**, arthropods trapped by the *Hymenaea* Defaunation resin are far from the Malaise traps. In **C**, arthropods trapped by the yellow sticky traps on *Agathis* tree trunks are far from the other yellow sticky traps on *Hymenaea* tree trunks. In **D**, Diptera trapped by the yellow sticky traps on *Agathis* tree trunks are far from all other yellow sticky and Malaise traps whether they were on or close to *Agathis* or *Hymenaea*. The prefixes ST and M stand for yellow sticky and Malaise traps, respectively, and R for resin (R1 collected in 2013, and R2 collected in 2015). ST followed by a number represents the height in metres at which the yellow sticky trap was placed on the tree trunk. T represents the sum of arthropods trapped in sticky traps in one location. The suffixes NC, MG, and MX stand for New Caledonia, Madagascar, and Mexico, respectively. Abbreviations: AAES: Amber from Ariño, Spain; AAFR: Amber from Archingeay-Les Nouillers, France, mentioned in Perrichot et al. [21]; AAIFR: Amber Aix island, France; AAPES: Amber from Arroyo de la Pascueta, Spain; ABFR: Amber La Buzinie, France; ACFR: Amber Cadeuil, France; ADCO: Amber Doumanga, Congo; ADOG.P: Dominican amber collection from the private collection mentioned in Poinar [53]; ADOJ.C: Dominican amber from the Jorge Caridad private collection; AFFR: Amber Fouras, France; AFTFR: Amber Fourtou, France; AHES: Amber from La Hoya, Spain; AMX: Mexican amber collections mentioned in Solórzano-Kraemer et al. [29]; APES: Amber from Peñacerrada I, Spain; ARFR: Amber Les Renardières, France; ASES: Amber from El Soplao, Spain; ASJES: Amber from San Just, Spain; ASFR: Amber Salignac, France; CDO: Copal collected in 2019 in Cotuí, Dominican Republic; CTZ: Copal from Tanzania; MMG: Malaise trap installed close to *Hymenaea* in 2013 in Madagascar; MMX: Malaise trap installed close to *Hymenaea* since 2010 to 2012 in Mexico; MNC: Malaise trap installed close to *Agathis* in 2016 in New Caledonia; RMG1: Defaunation resin collected from *Hymenaea* tree trunks in 2013 in Madagascar; RMG2: Defaunation resin collected from *Hymenaea* tree trunks in 2015 in Madagascar; RNC: Defaunation resin collected from *Agathis* tree trunks in 2016 in New Caledonia; ST0MG, ST1MG, ST2MG: Yellow sticky traps placed at 0, 1, and 2 m on *Hymenaea* tree trunks in Madagascar, respectively; ST0NC, ST1NC, ST2NC: Yellow sticky traps placed at 0, 1, and 2 m in 2016 on *Agathis* tree trunks in New Caledonia, respectively; STTMG: Total yellow sticky traps placed around *Hymenaea* in 2013 in Madagascar; STTMX: Total yellow sticky traps placed around *Hymenaea* tree trunks in 2012 in Mexico; STTNC: Total yellow sticky traps placed around *Agathis* tree trunks in 2016 in New Caledonia

The significant difference in arthropod trapping patterns observed in the Defaunation resins raises questions about the trapping efficiency of other entomological traps we used, which were placed: (i) on the trunk and (ii) close to the same tree species. These include yellow sticky traps, which resemble the sticky resin, and Malaise traps—both of which are used for arthropod collection and actuotaphonomic studies [29, 30, 51, 52]. Therefore, to better understand the trapping patterns and to assess whether *Agathis* tree resin traps arthropods in a similar way to yellow sticky traps, we analysed their arthropod assemblages using multidimensional scaling (MDS) with Bhattacharyya distance, in order to visualise the relationships between the taxa based on their frequency data. An important result is that the arthropods (at the order level) in the *Agathis* Defaunation resin, collected from the tree trunks in New Caledonia, plots far from the arthropods (at the order level) collected in both the yellow sticky and Malaise traps placed on the trunk and close to the *Agathis* trees, respectively (Fig. 4A) (in Additional file 3, we present the arthropod list). The arthropods (at order level) trapped in the yellow sticky traps in New Caledonia plotted far away from the arthropods trapped in the Malaise traps, but also far from the yellow sticky and Malaise traps from other sampling places, such as the *Hymenaea* forests in Madagascar and Mexico (Fig. 4C), where the *Hymenaea* trees were also sampled [29, 30]. In contrast, the samples from *Hymenaea* trees in Madagascar, analysed using the same method, plots the arthropods (at the order level) in the *Hymenaea* Defaunation resin from the tree trunks close to the arthropods collected in the yellow sticky traps (Fig. 4B).

Families in Diptera deserve a special analysis below the order level, attending to their high abundance in the collected material and in general in amber [54]. In the yellow sticky traps placed on the trunk of *Agathis*, Diptera made up more than 60% of the total. Within the Diptera, the family Phoridae comprised nearly 90% (Additional file 2: Fig. S2). At family level within Diptera, the yellow sticky traps placed on the trunk of *Agathis* from New Caledonia plot far away from all other samplings whether they were related to *Agathis* or *Hymenaea* (Fig. 4D).

The assemblage of trapped arthropods includes Acari, Collembola, Hemiptera, Thysanoptera, Hymenoptera, and Coleoptera, among other taxa (see Additional file 3 for arthropod list). The differences between flying and non-flying insects present in amber, copal, and Defaunation resin are not significant among the studied samples, except for the arthropods from the Malagasy Defaunation resin of *Hymenaea* and those from the Australian Eocene amber (Additional file 2: Fig. S3).

**Fig. 5:**
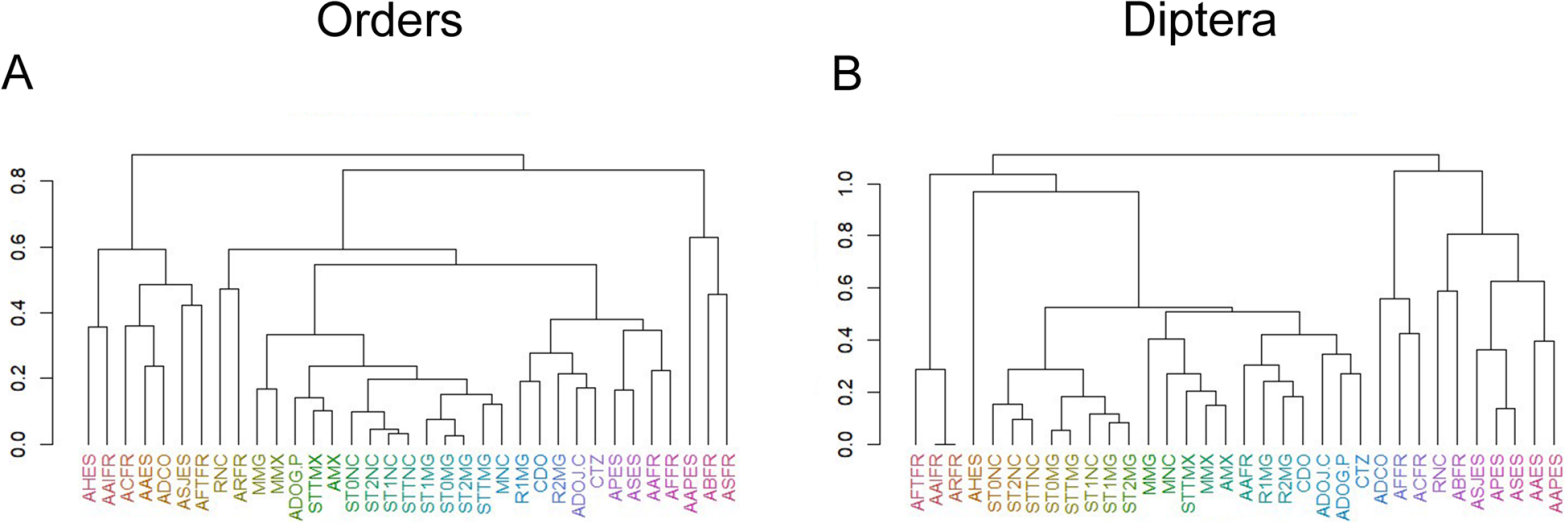
Dendrograms from hierarchical clustering of arthropods using 1-Spearman correlation at order and family levels for Diptera. **A** Arthropod specimens by orders in all collected samples of amber, copal, and Defaunation resin as well as arthropod specimens trapped by the yellow sticky and Malaise traps. **B** Dipteran specimens by families in all the collected samples of amber, copal, and Defaunation resin as well as dipteran specimens trapped by the yellow sticky and Malaise traps. To enhance interpretation, hierarchical clustering was applied to group similarly correlated variables, arranging them closer together, using 1-correlation distance and the complete linkage method. The meaning of the abbreviations is given in the Methods section

We also categorised the arthropods found in all the ambers, copals, and Defaunation resins originating from *Agathis* or *Agathis*-like trees and *Hymenaea* trees, and the arthropods trapped by yellow sticky and Malaise traps in the *Agathis* and *Hymenaea* forests (see Additional files 2 and 3 for details of the material and the lists of arthropods, respectively) using a hierarchical clustering (Fig. 5). *Agathis* and *Agathis*-like resins (Defaunation resins and ambers, respectively) cluster together at the order level and at family level in the case of Diptera (due to the high prevalence of the order Diptera in most samples, their families were analysed separately). Conversely, for New Caledonia, arthropods from both yellow sticky and Malaise traps placed on and close to the *Agathis* tree trunks, respectively, cluster together with those from amber, copal, and Defaunation resin derived from *Hymenaea*, as well as with the arthropods from both yellow sticky and Malaise traps on and close to the *Hymenaea* tree trunks.

As an additional result, we have updated the list of unbiased amber inclusions from various localities that can be used in future investigations (Additional file 3).

## Discussion

The number of amber localities lacking arthropod inclusions is unknown for most amber-bearing regions. This is due to the fact that palaeontological research focusses primarily on systematics and taxonomy, and on palaeoautecological information provided by the bioinclusions, in an attempt to reconstruct forest biocoenoses in deep time, while other aspects of the amber and amber deposits, such as taphonomy and geology, remain unstudied or poorly studied.

It is also important to mention that a large number of arthropod inclusions were yielded from some amber deposits discovered several years ago and worked for a long time. Peñacerrada I, for example, is notable for yielding the greatest number of arthropod inclusions from Spanish amber [18, 55]. Notably, it is also the locality from which the most amber has been extracted and prepared (see Table 1). The same is true for the Albian–Cenomanian amber locality from south-west France at Archingeay-Les Nouillers. Recently discovered deposits such as the Eocene amber from Australia or the Miocene amber from New Zealand, produced by *Agathis*, currently contain only a few arthropod inclusions [35, 41]. Thus, for some deposits, the absence of arthropod inclusions is most likely due to collection bias. However, it is clear that the majority of the Cretaceous amber deposits does not contain arthropod inclusions or are extremely poor [5]. Some authors have suggested that this circumstance for many Cretaceous amber deposits may be explained by a much more abundant resin exudation under confined conditions in the underground than in aerial conditions [49]. Araucariaceae trees, particularly *Agathis* spp., are known for their comparatively abundant production and exudation of resin, not only on their tree trunks, but also within their root systems [3, 37, 56–58]. In contrast to the hundreds of arthropod bioinclusions described from Malagasy copal and Defaunation resin [39], the copals found in regions like New Zealand and New Caledonia (own field. obs.) lack such abundant inclusions, and no arthropod bioinclusions have been described from this material. This can be partly explained by the fact that root resin is unlikely to trap arthropods in confined conditions; given the abundant production of root resin and the resulting mixture of aerial and root copal in these geological deposits, a large proportion of the collected pieces are of the root type. When examining the extant roots of *Hymenaea* trees and their resin production, a distinct contrast is evident (Additional file 2: Fig. S4). The resin in *Hymenaea* roots appear intricately interspersed in the sand, forming a crust around the roots ( [39] Fig. 8). In Madagascar, we observed large *Hymenaea* trees that had been uprooted by hurricanes, showing their root systems with only small amounts of resin, unlike large resin lumps commonly formed by *Agathis* roots [58]. In New Zealand, we have also had the opportunity to study the root systems of large Pleistocene *Agathis* at Waipapakauri [58] and Baylys Beach localities. These large trees are exhumed for their trunk wood, but the roots are abandoned, allowing us to observe large amounts of copal associated with roots (Additional file 2: Fig. S4C, D, and E).

To explain the exudation of resin on the tree trunk and the capability to trap arthropods, it is important to note that resin exudation, in general, archives a variety of possible defensive actions, for example, to shield trees from threats such as bark beetles and herbivores, as well as to facilitate wound healing, preventing fungal and bacterial infection, or to prevent desiccation [2, 5, 57, 59, 60]. The variation in resin composition plays a prominent role in those defensive actions. In performing those defensive actions, *Hymenaea* resin has been also demonstrated to accidentally act as a kind of entomological trap, more precisely as yellow sticky traps that works better for some arthropod groups than for others [29, 30]. Consequently, scientific interest in amber, copal, and Defaunation resin has been primarily focussed on arthropod inclusions [13]. However, the absence or fewer number of arthropod inclusions in many amber deposits, especially those of *Agathis* or *Agathis*-like origin, has never been questioned.

The very few arthropod inclusions in *Agathis* Defaunation resin compared to the large number of arthropod inclusions in *Hymenaea* Defaunation resin, along with the different values of exponent *b* (measure of distribution uniformity) for each resin assemblage, shows that *Hymenaea* resin is more efficient at trapping arthropods than *Agathis* resin and does so spatially more uniformly (see also Additional file 2: Fig. S1 and extended material and methods in Additional file 2). This is consistent with our observations in amber. All arthropod inclusions recorded so far in amber originated from *Agathis* or *Agathis*-like trees were found in a few pieces/lumps (non-uniform), whereas arthropod inclusions in amber originated from *Hymenaea* trees are more uniform (Additional file 2: Fig. S1), which means that there are more pieces with at least one inclusion.

The fewer arthropod inclusions and their non-uniform distribution in *Agathis* and *Agathis*-like resins compared with those observed in *Hymenaea* resin can be explained mainly through two different aspects, namely (1) resin composition and (2) arthropod attraction or repulsion, hereafter discussed more in detail.

### Resin composition

The variation in resin chemical composition between plant species affects the physical properties of the different resins, namely viscosity or stickiness, and consequently the capability to trap arthropods [28, 34, 37, 61].

Resin is a complex mixture of volatile compounds, including mono- and sesquiterpenoids, diterpenoids, and sometimes triterpenoids. The diterpenoids originate mainly from conifers (gymnosperms), while triterpenoids (e.g. of the oleanane, ursane, and lupane series) and sesquiterpenoids come from angiosperms [62]. These compounds contribute to the fluidity (sesquiterpenoids) of the resin and determine also its grade of viscosity (diterpenoids) [34]. *Agathis* and *Hymenaea* resins differ in their chemical composition. *Agathis* produces a resin type primarily composed of terpenoids [63]. In contrast, *Hymenaea* produces a resin containing both terpenoids and gum components [37]. The hardening and polymerisation of resin hinge on the number of free radicals within non-volatile compounds, particularly labdatriene diterpenoids, abundant in *Agathis* species [34, 64, 65].

**Fig. 6:**
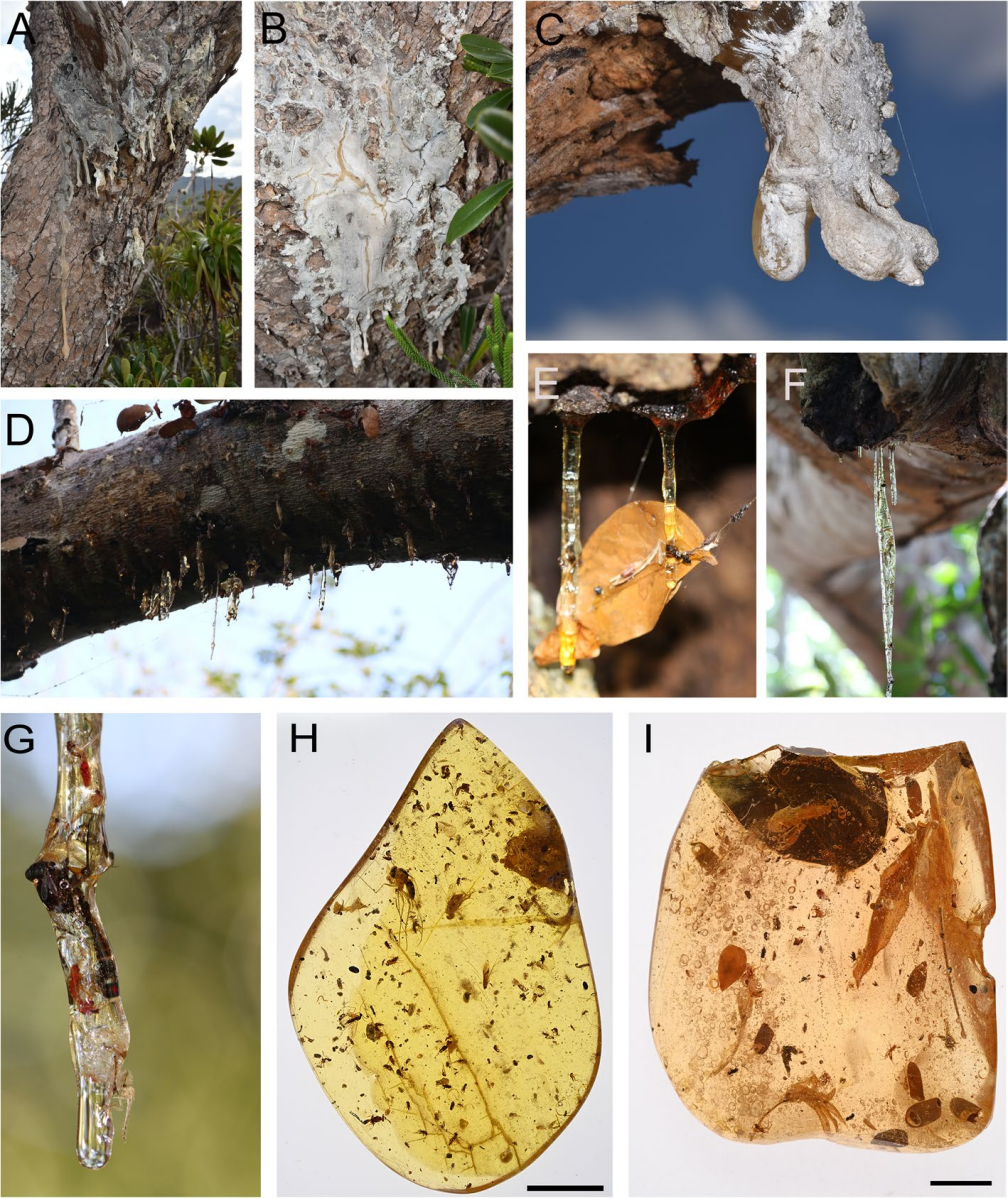
Resin (Defaunation resin) exuded by tree trunks and branches some with examples of trapped biological remains, principally arthropods and plants.** A**, **B**, and **C** *Agathis ovata* (C. Moore ex Veill.) Warb., 1900 in Col de Yaté, New Caledonia. **D**, **E**, and **F** *Hymenaea verrucosa* Gaertner, 1791 in the Mananjary region, Madagascar. Note the surfaces of the *Agathis* resin lumps with a whitish patina due to faster drying and hardening which prevent the presence of organismal inclusions (**A**, **B**, and **C**). *Hymenaea* resin, which generally is rich in arthropods and plant inclusions (as more clearly shows the photographs **H** and **I**), remains sticky for a long time and does not show a such whitish patina (**D**, **E**, and **F**), even after complete hardening/drying. **G** Trapped arthropods partially embedded in *Hymenaea* resin in the Mananjary region, Madagascar. **H** and **I** Trapped arthropods totally embedded in *Hymenaea* resin from the Mananjary region, Madagascar. Scale size in **H** and **I** is 10 mm

The effectiveness of resin as a defence mechanism under biotic and abiotic environmental stress depends on factors such as drying, flow rate, and viscosity. These factors determine, for example, whether arthropods are pushed out or trapped on the tree trunk, whether a microbial infection can be stopped, or whether desiccation can be prevented. In general, our observations show distinct drying and viscosity characteristics for *Agathis* and *Hymenaea* resins. This suggests that the faster polymerisation of *Agathis* resin, resulting in rapid drying (see Fig. 6C), likely leads to fewer arthropods being trapped. Conversely, the characteristics of *Hymenaea* resin imply an abundant trapping of biological remains (Fig. 6D–I). According to our observations, both resins are fluid at the time of exudation, *Agathis* even more than *Hymenaea*. However, this changes quickly as *Agathis* resin dries faster than *Hymenaea* does*.* Whether an arthropod gets stuck depends on it passing by just as the tree begins to produce resin and the organism walking or flying close enough to be trapped. It is quite likely that the fast-drying nature of *Agathis* resin prevents arthropods from sticking to it (Figs. 1 and 6).

The previous idea aligns with the sensitivity of *Agathis* spp. trees to the *Phytophthora* spp., a water- and soil-borne oomycete primarily affecting these tree species. This sensitivity prompts a substantial production and exudation of resin as a defence mechanism [3, 5, 66]. In consequence, the trees, both their trunks and roots, are producing large amount of resin. It has been hypothesised that rapid resin hardening may prevent the rapid spread of pathogens on the trunk of a tree [37, 67, 68]. However, *Agathis* is also attacked by the defoliating coccids, trips and boring beetles, among others [69]. Therefore, resin exudation in *Agathis* is not always induced by a pathogenic agent.

On the other hand, in most angiosperms, including *Hymenaea*, the resin is mixed with gum (a mixture of hydrophilic polysaccharides), which increases the water-holding capacity of the tissues and prevents desiccation [37, 57, 63]. Gum is rarely observed in conifers and not found in *Agathis* spp. [32]. The hydrophobic nature of gum increases the stickiness of the resin, as gum can form bonds and stick when it comes in contact with oily surfaces [70]. This may partly explain why the resins produced by some angiosperms remain sticky for a long time.

How quickly the resin dries depends mainly on the polymerisation rate (low polymerisation rate implies a liquid and sticky resin for a long time), and how long the resin can trap arthropods depends on how long it takes to dry. Therefore, regardless of the amount of resin exudation, the resin will trap arthropods on the entire surface of the accumulated resin for a longer time, making the distribution of arthropod inclusions more uniform (Additional file 2: Fig. S1B), as is the case of *Hymenaea* resin (Fig. 6D–I). A resin with a high polymerisation rate will remain fluid and sticky for a shorter period of time, giving arthropods a shorter period to be trapped in the resin, resulting in a non-uniform distribution (Additional file 2: Fig. S1C) of arthropod inclusions.

### Arthropod attraction or repulsion to resin

Attraction or repulsion toward resins may be physical or chemical in nature and are well documented for some arthropods [40]. From a physical point of view, the “water-imitating” reflection polarisation of resin has been proved for the presence of aquatic adult insects in amber [71]. Although more research is needed to understand how the light reflection of the resin may attract or repel other arthropods, an opaque and white surface (Figs. 1 and 6A, B, and C) may reflect less light than the transparent glue-like resin (Figs. 1 and 6D, E, and F), making *Agathis* resin a candidate for being less attractive.

From a chemical point of view, it has been well documented in some resin-producing conifers that (—)-pinene, a constituent of stem oleoresin, increases in response to heightened insect activity, suggesting a defence mechanism [60]. Abundant —(α)-pinene has also been reported in araucariacean resins [72]. Conifer oleoresin is a complex compound comprising various monoterpenes, sesquiterpenes, and diterpene resin acids. The turpentine portion of the oleoresin includes over 30 monoterpenes and many sesquiterpenes, serving as a defence mechanism by being toxic to some pathogens and insects. Additionally, it aids in sealing plant wounds through the hardening action of diterpenes [60]. Also, the labdanes have been a subject of research due to their potential use as natural insecticides, showcasing antifeedant properties against some Coleoptera, Diptera, and Lepidoptera [73]. Labdanes are diterpenes featuring two aromatic rings in their structure and are notably abundant in resins derived from the Araucariaceae [74, 75].

Volatile terpenoids, including labdanes, play then a dual role in defence by directly deterring herbivores and indirectly attracting their natural predators [76]. Moreover, these compounds also contribute to attracting pollinators for some gymnosperms such as cycads [77]. In the case of *Hymenaea*, both in Africa and South America, different resin compounds such as caryophyllene or α-humulene are present to defend against various caterpillars and termites [40]. The resin of some angiosperm species also attracts insect pollinators or animals (birds and mammals) that can disperse their fruits [57]. The attractiveness or repulsiveness of the resin is an important taphonomic bias in the trapping of some groups of arthropods. However, determining whether *Hymenaea* can attract arthropods in more abundance than *Agathis*, or primarily some particular arthropod groups, requires experimental investigation.

### The role of yellow sticky and Malaise traps in studying *Agathis* resin

Different orders of arthropods typically exhibit varying proportions in assemblages within amber, copal, and Defaunation resins, with Diptera, Hymenoptera, and Coleoptera being the most abundant orders. The type of organism present and its abundance are contingent upon several taphonomic and ecological variables [1, 3, 40], and these determined which part of the resiniferous forest is represented in the amber record [30]. In this context, we address two primary aspects of arthropod trapping: (1) whether *Agathis* resin traps arthropods in a similar way to yellow sticky traps, as observed with *Hymenaea* resin [30], and (2) whether the arthropod assemblage preserved in *Agathis* Defaunation resin is representative of the fauna in the same forest.

From our actuotaphonomic studies in Madagascar, we know that the arthropod assemblage in the *Hymenaea* Defaunation resin is comparable to that in the yellow sticky traps placed on *Hymenaea* trunks. We also know that the assemblage differs notably from the arthropod fauna trapped in Malaise traps placed close to the trunks. This means that the arthropod assemblages trapped by *Hymenaea* resin represent mainly the fauna living in and around the trunk [30]. This pattern was reinforced by our second sampling in Sacaramy, Madagascar ( [39] Fig. 1), and shown in Fig. 4B, in which MDS also plots the arthropod assemblages in Defaunation resin from *Hymenaea* close to the arthropod assemblages in yellow sticky traps. On the contrary, MDS plots arthropod assemblages (at order level) in resin from *Agathis* far away from the arthropod assemblages in yellow sticky and Malaise traps in *Agathis* (Fig. 4). This stark contrast shows that *Agathis* resins exhibit a different trapping bias as an entomological trap like the yellow sticky traps.

We found that arthropods preserved in amber and in Defaunation resin from *Agathis* and *Agathis*-like trees form distinct clusters at both the order and family levels (Fig. 5) (the latter focussed on dipteran families herein). In contrast, arthropods from yellow sticky and Malaise traps that were placed on and near *Agathis* trees cluster with those from amber, copal, and Defaunation resin of *Hymenaea* origin. They also cluster with the arthropods from yellow sticky and Malaise traps on and near *Hymenaea* trees. This reinforces the idea that the resin of *Hymenaea* acts as an entomological trap (i.e. it has a trapping effect similar to yellow sticky traps), and that there is difference in the ways *Agathis* and *Hymenaea* resins trap arthropods.

The arthropods trapped in *Agathis* Defaunation resin are mostly Arachnida or non-flying Hexapoda, except for one Thysanoptera and one Diptera remain. In contrast, the samples of Defaunation resin collected from *Hymenaea* tree trunks contain abundant Diptera (Additional file 3). However, as presented in the results, there was no a representative difference between flying and non-flying insects found in amber, copal, and Defaunation resin across the studied samples. Therefore, it is likely that this finding is due to the scarcity of material and needs more investigation.

The number of Diptera specimens was very high in the yellow sticky traps placed on *Agathis*; this may be a reason why they plot close to the Malaise traps (Fig. 4C and Additional file 2: Fig. S5C), since Malaise traps are considered a successful method to collect flies [78]. Within the Diptera, the family Phoridae overwhelmingly dominated the yellow sticky traps placed on *Agathis*, comprising nearly 90% of dipterans (Additional file 2: Fig. S2). As our collection took place in November and December, the humid months in New Caledonia, seasonality probably played an important role in determining abundance. Phoridae typically exhibit higher numbers during humid periods [79]. While other dipterans, namely Chloropidae, Sciaridae, and Cecidomyiidae, were also abundant in the yellow sticky traps placed at 1 m height, their numbers are small in comparison to Phoridae. This may be a reason why they plot separately from Malaise traps (Fig. 4D and Additional file 2: Fig. S5D). Although not as abundant as in yellow sticky traps on *Agathis*, the family Phoridae was also abundant in yellow sticky traps placed on *Hymenaea*, and this family is also abundant in amber, particularly in Miocene ambers (e.g. [80]). Phoridae flies can be collected using a variety of traps [29, 81, 82]; however, yellow sticky traps seem to be the most effective one for collecting these flies [83, 84]. Small vertebrates, such as lizards, can also become trapped in yellow sticky traps, drawing in phorid flies that are attracted to decaying animal matter [51, 83, 85]. This phenomenon may also partly account for the high abundance of dipterans, particularly phorid flies, observed in New Caledonia in yellow sticky traps. Surprisingly, Phoridae is not present in *Agathis* Defaunation resin, and Diptera is notably underrepresented in that resin (Additional file 3). Resin production is affected by various factors, including temperature, humidity, growth, carbon assimilation, soil nutrients, or injuries [3, 31]. Therefore, it is not possible to establish a correlation between seasonality and the absence of phorid flies in the resin.

## Conclusions

Given that *Agathis* and *Hymenaea* resins trap arthropods differently, a pattern inferred for extant and deep-time ecosystems, it is crucial to consider these different trapping biases (Fig. 1) when comparing fossil assemblages preserved in ambers from these two botanical sources.

Several important factors influence the trapping of arthropods by *Agathis* resin. We consider that the most important factor is rapid drying, which results in a higher viscosity and the acquisition of a whitish patina on resin exuded from the trunk—even though *Agathis* resin is much more fluid than *Hymenaea* resin when it is originally exuded. Rapid drying prevents a large number of arthropods from being present as bioinclusions in the *Agathis* Defaunation resin, and it results in a statistically non-uniform spatial distribution of these. In contrast, *Hymenaea* resin has a higher viscosity when exuded, but it takes much longer to dry, and remains transparent; the slower drying allows arthropods to be abundant as bioinclusions in the resin, making their spatial distribution statistically more uniform. This leads us to conclude that *Agathis* resin does not act as an effective entomological trap for arthropods, unlike *Hymenaea* resin (Fig. 1). This suggests that the arthropods trapped in *Agathis* Defaunation resin (thanatocoenosis) (which eventually become amber—oryctocoenosis) do not reflect the fauna that lived in or surrounding the resin-producing trees (biocoenosis), unlike *Hymenaea* resin. This taphonomic pattern has critical implications for the interpretation of Cretaceous forest biocoenoses with an *Agathis* or *Agathis*-like origin. Although we discuss that *Agathis* resin is less effective at trapping arthropods than *Hymenaea* resin, we could not determine frequent ecological groups (arthropod guilds) trapped by *Agathis* resin using the small sample set that is available. Similarly, the fossil assemblages of arthropod bioinclusions in Eocene or Miocene *Agathis* ambers do not provide guild information due to the scarcity of deposits. A follow-up investigation (beyond collecting more *Agathis* resin with bioinclusions) could involve defining the criteria required to recognise guilds and categorising the inclusions found in Cretaceous amber, in order to determine the most prevalent types of arthropods in this amber type.

The abundance of Cretaceous amber and Quaternary copal localities featuring numerous lumps but few bioinclusions, along with the non-uniform distribution of rare arthropod inclusions, can be partly attributed to substantial resin exudation from *Agathis* root systems. This exudation forms both large and small lumps in confined conditions. Comparable profuse resin production has not been observed from the root system of *Hymenaea* trees. Several unanswered questions still require further investigation, such as how resin attracts or repels some arthropods influencing the number of arthropods trapped, and the content of bioinclusions in the oryctocoenosis. Furthermore, this influence needs to be considered with respect to whether the trees involved were from the genera *Agathis* or *Hymenaea*, or from some close relatives.

Further studies and more accurate taphonomic data are needed to identify the conditions in which arthropods were trapped in other ancient and modern resins from coniferous and leguminous trees, and to determine and how the oryctocoenosis originated.

## Methods

### Material

We collected living arthropods using yellow sticky and Malaise traps, following the methodology outlined by Solórzano-Kraemer et al. [30]. These traps were installed on and close to *Agathis lanceolata* (Lindl. ex Sebert & Pancher) Warb., 1900 trees in Bon Secours forest, adjacent to the Rivière Bleue Provincial Park (New Caledonia), in 2016 (November–December, warm and rainy season), and on and close to *Hymenaea verrucosa* in Madagascar, Mananjary region (between Nosy Varika and Ambahy), in 2013 (September–October, warm and rather dry season), and Sacaramy (near to Diego Suarez), in 2015 (April–May, warm and rainy season) (Additional file 2: Fig. S6 and extended material and methods in Additional file 2). In total, we collected arthropods around four *Agathis* trees and eight *Hymenaea* trees (four *Hymenaea* trees per campaign). During each collection trip, we displayed 45 yellow sticky traps at three different heights on each tree for 8 days, as well as four Malaise traps, one for each of four selected trees. We added to our analyses the data extracted from Solórzano-Kraemer et al. [29], who also collected arthropods with yellow sticky and Malaise traps in 2010, 2011, and 2012 on and close to *Hymenaea courbaril* Linné, 1753 in La Rinconada National Park in Mexico. The list of sampled arthropods can be seen in Additional file 3.

Throughout the manuscript, we use the terms amber, copal, and Defaunation resin sensu Solórzano-Kraemer et al. [13]. In this system, amber is older than 2.58 Ma, copal is 2.58 Ma to 1760 AD (Anno Domini), and Defaunation resin is the resin produced after 1760 AD.

We sampled Defaunation resins from eight *Agathis lanceolata* trees and from 28 *A. ovata* trees in New Caledonia. In Madagascar, resin was collected from 11 *H. verrucosa* trees in the Mananjary region in 2013 [30] and from 10 *H. verrucosa* trees in Sacaramy (for the precise location and map see [39], Fig. 1) in 2015. All Defaunation resin samples were collected from tree trunks. From Mexico, no Defaunation resin was sampled from *Hymenaea courbaril* trees because resin exudates were virtually absent [29].

For the purposes of this paper, we do not distinguish between the different extant species of *Agathis* and *Hymenaea* involved in the collection work, or between the different ancient tree species proposed as the origin of the resin in the past (in the case of amber derived from *Hymenaea*, e.g. *H. protera*† Poinar, 1999, *H. mexicana*† Poinar and Brown, 2002, and *H. allendis*† Calvillo-Canadell et al., 2010). We use “*Agathis*” to refer to both living trees and fossil resins derived from *Agathis* trees. “*Agathis*-like” refers to fossil resins derived from ancient plants of the genus *Agathis* or only related to it. Cheirolepidiaceous ambers are also included as they are highly similar in chemistry to *Agathis* resins [8, 47]. Since the proposed botanical origin of Miocene amber is not questioned, we use *Hymenaea* to refer to both living trees and resins, as well as fossil resins derived from *Hymenaea*.

We compared the arthropod assemblages obtained from yellow sticky and Malaise traps and those preserved within Defaunation resin from extant trees, with the arthropod assemblages preserved in amber from Late and Early Cretaceous and Middle Miocene from different localities around the world. All the amber, copal, and Defaunation resin collections included in the present study have been selected because they are unbiased and collected for scientific purposes; they have been collected in the field without prior selection of arthropod inclusions and/or whether the pieces/lumps containing arthropod inclusions or not. Searching for arthropod inclusions and preparing the pieces is a process that has been carried out in laboratory. With this purpose, we persuaded a detailed composition of the arthropod assemblages identified from Cretaceous amber from Spain and France, Eocene amber from Australian, Oligocene and Miocene amber from New Zealand, Miocene amber from the Dominican Republic and Mexico, and copal and Defaunation resin from the Dominican Republic. Collections with the specification of the inclusions per gram of resin are mentioned in Table 1.

The term bioinclusion in amber, copal, and Defaunation resin includes all organismal remains that can be found in fossil resins. However, since arthropod inclusions are the only bioinclusions included in our analyses, we will use the term arthropod inclusions to refer to the bioinclusions we study here.

The copal and Defaunation resin samples were polished and prepared in the same manner as amber [86, 87] to correctly identify the bioinclusions. However, for some lumps, it was only necessary to create a small viewing window to observe their contents.

### Imaging

The photographs were performed with digital cameras Canon EOS 40D and Canon EOS 70D. Figures were performed using Adobe Photoshop software (version 25.4 www.adobe.com).

### Data processing

The data used in this study were frequency data of taxa and experimental groups (amber, copal, and Defaunation resin, and yellow sticky and Malaise traps). Multivariate analyses were used to evaluate the different hypotheses of the study, namely, multidimensional scaling (MDS), and representation of the relationships for each experimental group in the form of a correlation heat-map or correlation network.

Statistical analyses were performed using different R functions and libraries [88]. The BDbiost3 library for R [89, 90] was used for exploratory data analysis, and multidimensional scaling used functions LinesMDS() (see Additional file 2 for more detail on the statistical methods used [91, 92]). All data used for statistical work are available in Additional file 3.

To estimate the degree of uniformity of arthropod inclusions in Defaunation resin from *Agathis* and *Hymenaea*, we estimated the probability density function (PDF) that a piece lacks arthropod inclusions given the mass of the piece (see Additional file 2 for a discussion of this methodology [93]). For this, we measured the mass of pieces with an error of 0.01 g and we counted the number of arthropod inclusions in each piece (see Additional file 3). Then, we calculated the frequencies of pieces without arthropod inclusions and with a mass in intervals of 0.01 g for the Defaunation resins from the *Agathis* and *Hymenaea* trees. These frequencies were associated with the conditional probability of finding a piece of mass *m*, given that it does not contain arthropod inclusions. Bayes’ theorem was then used to obtain the conditional probability of not having arthropod inclusions given that the piece has mass *m*. Finally, we fit the resulting probabilities with power laws of the form $a{m}^{-b}$ by first obtaining the logarithm of the data, then using standard least squares algorithm with the fitting model log(*a*) − *b* log(*m*) to obtain the values of *a* and *b* for both collections. We used *b* as a measurement of the uniformity of arthropod inclusions. The exponent *b* measures the uniformity of arthropod inclusions, while the parameter *a* is just the estimated value of the PDF that a piece of resin has no arthropod inclusions within 1 g of resin, and was only used to fit the data correctly.

## Supplementary Information

Additional file 1. Abstract in Spanish.

Additional file 2. Extended Material and Methods and Supplementary Figures S1 to S6. Fig. S1 – Theoretical distributions of bioinclusions in a piece of resin. Fig. S2 – Diptera families entrapped around and close to Agathis lanceolata by yellow sticky traps at 0, 1, and 2 meters high, and by Malaise traps, respectively. Fig. S3 – Mosaic plot analysis to represent the results of the observed relative frequencies between deposits and flying and non-flying insects. Fig. S4 – Roots of resin-producing trees. Fig. S5 – Multidimensional scaling (MDS) showing the vectors of influence using Bhattacharyya distance for arthropod orders (A–C) and Diptera families (D) trapped on the trunk and close to Agathis and Hymenaea trees. Fig. S6 – Map showing the localisation of New Caledonia and the sampling localities cited in the text.

Additional file 3. List of unbiased amber inclusions from various localities.

Additional file 4. Arthropod inclusions per gram of resin.

## Data Availability

All the data needed to evaluate the conclusions of the paper are available in the paper and in the supplementary information files. The amber from “El Valle – 7 Cañadas” and San Rafael, Dominican Republic have been acquired directly in the mine by MMS-K, XD and EP. The copal/Defaunation resin from Cotuí, Dominican Republic, have been acquired by Y.H. Shih. These amber and copal/Defaunation resin will be housed in the Museo Nacional de Historia Natural ‘Prof. Eugenio de Jesús Marcano’ in Santo Domingo, Dominican Republic. Correspondence for material related to this paper can be sent to Mónica M. Solórzano-Kraemer (monica.solorzano-kraemer@senckenberg.de), Enrique Peñalver (e.penalver@igme.es), and Xavier Delclòs (xdelclos@ub.edu).
