# Supplementary material for: Agathis vs. Hymenaea—trapping biases to interpret arthropod assemblages in ambers
Source: BMC Biol. 2025 Nov 7;23:337. doi: 10.1186/s12915-025-02453-y (PMC12593905; doi:10.1186/s12915-025-02453-y)
Supplement: Supplementary file 1 — Additional file 1. Abstract in Spanish. [file 12915_2025_2453_MOESM1_ESM.docx]

This translation in spanish was submitted by the authors and we reproduce it as supplied. It has not been peer reviewed. Our editorial processes have only been applied to the original abstract in English, which should serve as reference for this article. This translated abstract is published under the same licence as the article.

**Antecedentes** Los géneros *Agathis* (Coniferales: Araucariaceae) e *Hymenaea* (Fabales: Fabaceae) incluyen especies de árboles productores de resina cruciales para estudios actuotafonómicos. Mientras ciertos ámbares del Cretácico probablemente se originaron de árboles *Agathis* o afines, *Hymenaea* es la fuente principal de muchos ámbares del Mioceno. Para este estudio, se realizaron trabajos de campo en Nueva Caledonia y Madagascar para recolectar resina de Defaunación (resina producida después de 1760 d.C.). Se recolectaron artrópodos con trampas adhesivas amarillas y trampas Malaise en Nueva Caledonia, Madagascar y México. Se analizaron ámbares del Cretácico y del Mioceno, copales (desde hace 2,58 Ma a 1760 d.C.) y resinas de Defaunación de diversas regiones para comparar los patrones de captura de artrópodos.

**Resultados** Los resultados actuotafonómicos muestran un menor número de artrópodos atrapados en la resina de Defaunación de *Agathis*, con distribución no uniforme, en comparación con los artrópodos abundantes y uniformemente distribuidos en la resina de Defaunación de *Hymenaea*. El menor número de inclusiones en la resina producida en los troncos de *Agathis* se atribuye a su rápida polimerización. En las mismas condiciones experimentales, los artrópodos de la resina de Defaunación de *Agathis* difieren de los capturados en trampas adhesivas amarillas y Malaise, mientras que los de la resina de Defaunación de *Hymenaea* se asemejan a los de las trampas adhesivas amarillas.

**Conclusiones** Estos hallazgos confirman los distintos patrones de captura de resina entre *Agathis* e *Hymenaea*, con importantes implicaciones para la interpretación del registro fósil en ámbar. La fauna atrapada por la resina de *Hymenaea* se asemeja a la biocenosis de artrópodos que habita en el tronco y su entorno, lo que indica autoctonía y una estrecha relación con el ecosistema forestal, a diferencia de la fauna atrapada por la resina de *Agathis*. Estos resultados mejoran la comprensión de los sesgos de captura de artrópodos en resina y llevan a reconsiderar las interpretaciones previas sobre las biocenoses forestales del Cretácico.
