# Supplementary material for: Agathis vs. Hymenaea—trapping biases to interpret arthropod assemblages in ambers
Source: BMC Biol. 2025 Nov 7;23:337. doi: 10.1186/s12915-025-02453-y (PMC12593905; doi:10.1186/s12915-025-02453-y)
Supplement: Supplementary file 2 — Additional file 2. Extended Material and Methods and Supplementary Figures S1 to S6. Fig. S1 – Theoretical distributions of bioinclusions in a piece of resin. Fig. S2 – Diptera families entrapped around and close to Agathis lanceolata by yellow sticky traps at 0, 1, and 2 meters high, and by Malaise traps, respectively. Fig. S3 – Mosaic plot analysis to represent the results of the observed relative frequencies between deposits and flying and non-flying insects. Fig. S4 – Roots of resin-producing trees. Fig. S5 – Multidimensional scaling (MDS) showing the vectors of influence using Bhattacharyya distance for arthropod orders (A–C) and Diptera families (D) trapped on the trunk and close to Agathis and Hymenaea trees. Fig. S6 – Map showing the localisation of New Caledonia and the sampling localities cited in the text. [file 12915_2025_2453_MOESM2_ESM.docx]

**Supplementary Information for**

***Agathis* vs. *Hymenaea* - trapping biases to interpret arthropod assemblages in ambers**

**Mónica M. Solórzano-Kraemer*^1^, Antonio Monleón-Getino^2^, Enrique Peñalver^3^, Atahualpa S. Kraemer^4^, Mélanie C. M. Herbert^1^, David Peris^5^, Antonio Arillo^6^, Vincent Perrichot^7^, Eduardo Barrón^8^, Romain Garrouste^9^, Maria Paulsen^10^ & Xavier Delclòs^11, 12^**

^1^Senckenberg Research Institute, 60325, Frankfurt am Main, Germany

^2^BIOST^3^. GRBIO. Section of Statistics, Department of Genetics, Microbiology, and Statistics, Faculty of Biology, University of Barcelona, 08028 Barcelona, Spain

^3^Instituto Geológico y Minero de España, IGME-CSIC, 46004 Valencia, Spain

^4^Departamento de Física, Facultad de Ciencias, Universidad Nacional Autónoma de México, Ciudad Universitaria, 04510, Mexico City, Mexico

^5^Institut Botànic de Barcelona (CSIC-CMCNB), 08038, Barcelona, Spain

^6^Departamento de Biodiversidad, Ecología y Evolución, Facultad de Biología, Universidad Complutense, 28040, Madrid, Spain

^7^Géosciences Rennes (UMR 6118), Univ Rennes, CNRS, 35000 Rennes, France

^8^Instituto Geológico y Minero de España, IGME-CSIC, 28003 Madrid, Spain

^9^Institut de Systématique, Evolution et Biodiversité, UMR 7205 – CNRS, MNHN, SU EPHE, Muséum National d'Histoire Naturelle, CP50 Entomologie, 75005 Paris, France

^10^School of Earth, Atmosphere, and Environment, Monash University, Clayton, 3800 Victoria, Australia

^11^Departament of Earth and Ocean Dynamics, Faculty of Earth Sciences, University of Barcelona, 08028 Barcelona, Spain

^12^Biodiversity Research Institute (IRBio), University of Barcelona, 08028 Barcelona, Spain

*e-mail: [monica.solorzano-kraemer@senckenberg.de](mailto:monica.solorzano-kraemer@senckenberg.de)

**Extended Material and Methods**

**Supplementary Figures S1 to S6**

**Supplementary References**

**Extended Material and Methods**

**Material**

**Amber.** The raw Miocene amber from the Dominican Republic used in this study is unbiased both in terms of the absence of pre-selection of arthropod species and in its representation of arthropods per weight. The amber comes from “El Valle – 7 Cañadas” and from “San Rafael” and was acquired directly from the mines. The amber from “El Valle – 7 Cañadas”, with a weight of 1,678.8 grams, contained 270 arthropod inclusions, while the amber from “San Rafael”, with a weight of 523.5 grams, contained 140 arthropod inclusions.

Dominican amber arthropod inclusions were identified as a part of the private collection of J. Caridad in Santo Domingo (Dominican Republic) housed at the World Amber Museum, with the owner's permission. The collection is unbiased only in that there is no pre-selection of arthropod species, and not in terms of arthropods per weight of amber. We also included the arthropod assemblages identified from Miocene amber from the Dominican Republic reported by Poinar [90]. Furthermore, we also added the results reported by Solórzano Kraemer et al. [29], who found 107 arthropod inclusions in 2,000 grams of raw Mexican amber.

The collections from Poinar [90], J. Caridad, and two collections from the Mexican amber from Solórzano-Kraemer et al. [29], do not have the information related to the weight of the whole sample because they were pre-selected for the presence or absence of arthropod inclusions. Thus, these collections are only used for the analysis of the type of arthropods as inclusion in diverse modern resins and fossil resins. Collections with the specification of the inclusions per gram of resin are mentioned in Table 1.

The Albian–Cenomanian amber from France comes from several deposits where the amber contains bioinclusions, namely Archingeay-Les Nouillers, Le Renardière, Fouras/Bois Vert, l’Ile d’Aix, Cadeuil, La Buzinie, Fourtou, and Salignac. The data used from the French amber comes from Perrichot [20] and Perrichot et al. [21], and V. Perrichot updated the list of arthropod inclusions with the data obtained until 2025. The amber is unbiased in that there is no pre-selection of arthropod species, and also in terms of arthropods per weight of amber.

The amber from Spain comes from seven deposits where the amber contains bioinclusions, namely Ariño, El Soplao, La Manjoya, Peñacerrada I, Arroyo de la Pascueta, San Just, and La Hoya. All of them are Albian to Cenomanian in age [5, 18, 83]. The amber is unbiased in that there is no pre-selection of arthropod species, and also in terms of arthropods per weight of amber. The list of arthropod inclusions has been updated by E. Peñalver, X. Delclós, A. Arillo and M.M. Solórzano Kraemer with the help of R. López del Valle

The Aptian amber from Congo comes from one deposit in which amber contains bioinclusions, namely Doumanga [84], and V. Perrichot updated the list of arthropod inclusions with the data obtained until 2025. The amber is unbiased in that there is no pre-selection of arthropod species, and also in terms of arthropods per weight of amber.

Eocene Australian amber data were extracted from Stilwell et al. [35], and updated by one of us (M. Paulsen), and Oligocene and Miocene New Zealand amber data were extracted from Schmidt et al. [45].

Bioinclusion tables have been collected over time and updated for this publication (Additional file 3). We included in the list the arthropod classes Arachnida, Chilopoda, Diplopoda, Malacostraca, Collembola, Diplura, and Insecta, which we identified in Defaunation resins and in the yellow sticky and Malaise traps. We identified the hexapods at the order level. Hemiptera and Lepidoptera have also been identified at the suborder level (Auchenorrhyncha, Sternorrhyncha, and Heteroptera, and Glossata and Zeugloptera, respectively). The order Diptera has been identified at the family level, as this order dominates in the amber, copal, and Defaunation resin assemblages, and also in the yellow sticky and Malaise trap samples.

**Copal/Defaunation resin from the Dominican Republic.** Copal/Defaunation resin from Cotuí (Dominican Republic) was collected from the soil in pineapple plantations and therefore unbiased in that there is no pre-selection of arthropod species, and also in terms of arthropods per weight of copal/Defaunation resin fragments/lumps. The copal/Defaunation resin was sampled in 2019 and has been polished and prepared in the same way as amber [86]. In Cotuí, no *Hymenaea* trees were found. Plant remains (other type of bioinclusions) were also present, but were excluded from the statistical analysis. The age of each piece was not analysed; however, some pieces from the region have been dated as young 160 BP and as old 36,000 BP [13].

**Defaunation resin from Madagascar and New Caledonia**. Resins (Defaunation resins *sensu* Solórzano-Kraemer et al. [30]) from *A. lanceolata*, *A. ovata,* and *H. verrucosa* trees were studied for arthropod inclusions (bioinclusions). Standard methods in amber research were used, such as polishing resin to create visibility "windows" and cutting the resin piece to isolate bioinclusions, if necessary, for microscopic examination. The tree resin was collected at heights up to 4 meters from the ground. The study also differentiated Hemiptera into suborders due to their distinct biologies. Specimen sizes were measured under microscopes, and the minimum number of individuals was determined by the presence of the thorax/prosoma, except for termites and ants, which were counted differently. Termites were also counted if only the wings were present, and ants were also counted if only the heads were present.

Defaunation resin collected in Madagascar in 2013 and Defaunation resin collected in New Caledonia in 2016 are housed at the Instituto Geológico y Minero de España (IGME), CSIC, in Madrid, Spain. Additionally, resin collected in Madagascar in 2015 is housed at the Senckenberg Research Institute in Frankfurt, Germany.

**Resiniferous trees.** *Agathis lanceolata* was sampled in Bon Secours forest, close to the limit of the Rivière Bleue Provincial Park, situated in the South Province (Great South) of New Caledonia (Fig. S6). In this area, *A. lanceolata* lives under an Af climate (Köppen-Geiger climate classification), a hot, humid tropical climate with all months above 18°C. The average annual temperature is 24.3˚C; the temperature in October-December averages 22.5˚C. The average annual rainfall is 1,094 mm. The driest month is October, with 50 mm of rain. In March, the rain reaches its annual peak, with an average of 144 mm. The altitude of Rivière Bleue is around 200 meters above sea level, and the area is home to 131 species of woody plants, including the endemic gymnosperm *A. lanceolata*.

*Agathis ovata* was sampled in Col de Yaté, about 15 km west of the Rivière Bleue Provincial Park, within shrublands (maquis) in New Caledonia (Fig. S6). In this area, *A. ovata* lives under the same climate conditions as Blue River Provincial Park, but it is a little more humid because it is at a higher altitude (321 a.s.l.) and closer to the sea. Col de Yaté experiences a mean annual rainfall of 2,389 mm, with most of this rain occurring during the wet months from December to March. As in Madagascar, the area can be affected by hurricanes from the end of January to March. Fieldwork was also carried out at the onset of the rainy season.

*Hymenaea verrucosa* were sampled in the lowland forest on the North-East side of Madagascar, in the Mananjary region (between Nosy Varika and Ambahy, in 2013) and in Sacaramy (close to Antsiranana, Diego Suarez, in 2015). In the Mananjary region, *Hymenaea* is common in the lowlands near the sea (not on the coastline), at elevations of 5–10 meters above sea level, characterized by a subequatorial climate along the east coast (for more information, see [30, 39]). The trees grow in diverse habitats, including dry and wet evergreen forests, woodland, and evergreen bushland (de la Estrella, 2018). The average annual temperature is 23.1˚C (the variation in annual temperature is around 6.2˚C). The annual rainfall is high, averaging 2,439 mm, with rain even during the driest month. *Hymenaea* trees are used there to provide shade for the vanilla plants. Fieldwork was carried out at the onset of the rainy season when temperatures reached up to 31°C, high humidity of 80%, and rainfall as high as 3,500 mm.

In Sacaramy, in the Antsiranana region*, H. verrucosa* lives under a tropical savanna climate (Aw in Köppen-Geiger classification) (for more information see [39]). Rainfall is much more significant in summer than in winter. The average annual temperature is 26.5˚C; the temperature in December averages 28.0˚C. The average annual rainfall is 1,156 mm. The driest month is September, with 11 mm of rain. In January, the rain reaches its annual peak, with an average of 320 mm. Fieldwork was also carried out at the onset of the rainy season.

**Yellow sticky and Malaise traps.** The traps were deployed, and active capturing organisms, for eight days in all the collecting places. In each area, we sampled four trees with yellow sticky straps and one Malaise trap on each of four trees. Subsequently, traps were carefully removed, and the trapped specimens were transferred to clean containers containing 70% alcohol for preservation. Previously, for the yellow sticky traps, the process involved dissolving the glue from the yellow sticky sheet surfaces using petrol to release the trapped specimens. The specimens (mainly arthropods) from the yellow sticky traps were promptly transferred to containers with alcohol for preservation. Unfortunately, in New Caledonia, the yellow sticky sheets made of plastic dissolved in the petrol we used there. This was likely due to the different composition of the petrol sold in New Caledonia, as this method worked well in Madagascar and Mexico. For this reason, the samples obtained with yellow sticky traps from two trees in New Caledonia could not be studied. For a more detailed description of the methodology, refer to Solórzano Kraemer et al. [30].

Arthropods collected using yellow sticky and Malaise traps in Madagascar and New Caledonia are housed at the Senckenberg Research Institute in Frankfurt (**SMF**), Germany.

The amber, copal, and Defaunation resin, and the arthropods collected with the yellow sticky and Malaise traps mentioned in this work, are housed at the following institutions:

- Amber: Colección Institucional del Laboratorio de la Cueva El Soplao in Celis, Cantabria, Spain (**CES**); Museo de Ciencias Naturales de Álava, Victoria-Gasteiz, Spain (**MCNA**); Museum de la Universitat de València d’Història Natural, Burjassot, Valencia Province, Spain (**MUVHN**); Museo Aragonés de Paleontología (Fundación Conjunto Paleontológico de Teruel-Dinópolis), Teruel, Spain; Department of Geosciences of the University of Rennes (formerly the Geological Institute of Rennes), Rennes, France (**IGR**); Museo Nacional de Historia Natural ‘Prof. Eugenio de Jesús Marcano’ in Santo Domingo, Dominican Republic (**MNHNSD**); Private collection of J. Caridad housed at the World Amber Museum (Santo Domingo, Dominican Republic); Museums Victoria, palaeontology collection, Melbourne, Victoria, Australia (N**MV**); and Geology Museum, University of Otago, Dunedin, New Zealand (**OU**).
- Copal: Senckenberg Research Institute and Museum in Frankfurt am Main, Germany (**SMF**); and Museo Nacional de Historia Natural ‘Prof. Eugenio de Jesús Marcano’ in Santo Domingo, Dominican Republic (**MNHNSD**).
- Defaunation resins: Senckenberg Research Institut and Museum in Frankfurt am Main, Germany (**SMF**); and El Instituto Geológico y Minero de España (**IGME-CSIC**).
- Recent fauna from the yellow sticky and Malaise traps: Senckenberg Research Institut and Museum in Frankfurt am Main, Germany (**SMF**).

**Statistical methods**

The data used in this study were frequency data of taxa and experimental groups (amber, copal, and Defaunation resin as natural assemblages, and entomological traps as experimental assemblages). This section describes in more detail some of the statistical methods used.

**Multidimensional Scaling (MDS).** Multidimensional Scaling (MDS) is a statistical technique used to visualize relationships among a set of data points [90]. This is achieved by mapping the data points onto a lower-dimensional space, such as a two-dimensional plot. The primary goal of MDS is to find a representation that optimally preserves the dissimilarities (distances) between these data points. In this study, MDS was applied to visualize the relationships between the taxa based on their frequency data. For this analysis, three well-known dissimilarity measures [91], with which the research team has extensive experience, were employed to quantify dissimilarity between probability distributions, making them particularly suitable for count data: a) Bhattacharyya distance: quantifies the similarity between two probability distributions; b) Kullback-Leibler divergence: measures how one probability distribution diverges from a second, reference probability distribution and c) Bray-Curtis distance: assesses the dissimilarity between two samples based on the abundance of shared and unshared items.

The resulting MDS plots display axes (components) that are mathematically derived from the specific distance matrix used, with each axis indicating the proportion of variance explained by that component in the corresponding distance scaling.

In Multidimensional Scaling (MDS), the X and Y axes (e.g., Fig. 3) represent the coordinates of the data points in a newly constructed, lower-dimensional space, typically a two-dimensional plane. These axes are not predefined like in a standard Cartesian plot; instead, they are mathematically derived from the input dissimilarity matrix. The MDS algorithm takes the calculated distances (e.g., Bhattacharyya, Kullback-Leibler, or Bray-Curtis distances) between all pairs of data points. It then iteratively positions these points in the new, reduced-dimensional space (defined by the X and Y axes) such that the distances between the points in this new space optimally reflect the original dissimilarities. Each axis (or component) is orthogonal and is constructed to capture the maximum possible amount of the original dissimilarity structure. The "proportion of variance explained by each component" refers to how much of the original dissimilarity information is represented by that specific axis in the scaling derived from the chosen distance measure.

To interpret the plots further, a biplot was created to demonstrate the impact of taxa on sample distribution. This was achieved by overlaying vectors onto the MDS plot, with each vector representing a taxon. The direction and length of each vector indicate the correlation between the abundance of the taxon in question and the positions of the samples on the plot. This effectively illustrates which taxa are responsible for the separation between samples.

**Spearman's Correlation Analysis.** Spearman's correlation analysis is a statistical technique that is used to measure the strength and direction of the association between two variables. It is a non-parametric test, which means that it does not make any assumptions about the distribution of the data. In this study, Spearman's correlation analysis was used to assess the relationships between the taxa based on their frequency data. This analysis was used to identify pairs of taxa that were significantly more or less similar to each other than would be expected by chance. In this study, this type of correlation is used because the data is of a frequency type and not normal.

**Hierarchical cluster analysis.** Hierarchical clustering is a statistical method used to group a set of data points into a hierarchy of clusters, which is then visualised as a dendrogram. This technique was employed to assess the relationships between arthropod orders and Dipteran families based on frequency data. First, we used the Hmisc library to compute a Spearman correlation matrix. This was then converted into a distance matrix by subtracting the correlation coefficients from 1, a process known as 1-correlation distance. This ensures that highly correlated taxa have a small distance between them. The hclust() function was then used to perform the clustering using the complete linkage method to determine the distance between clusters. The dendextend library was then used to customise the visual appearance of the resulting dendrogram, enhancing its interpretability. The height at which branches merge in the dendrogram indicates dissimilarity between groups, with shorter branches signifying higher correlation and greater similarity in occurrence patterns.

**Mosaic Plot Analysis and Proportional Significance.** Mosaic plots were utilized, specifically using the mosaicplot() function, to represent the observed relative frequencies and visualize relationships between insect groups and their occurrence across various deposits. To complement these visual patterns, binomial statistical tests were employed using the binom.test() function to ascertain the statistical significance between proportions, thereby confirming the observed associations and differences.

**Regressions:** To fit the data of Figure 1, we used least squares over a power law of the form [
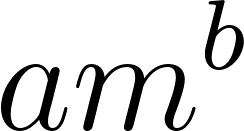
](https://www.codecogs.com/eqnedit.php?latex=a%20m%5E%20b#0), where m is the mass of the resin, [
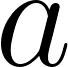
](https://www.codecogs.com/eqnedit.php?latex=a#0) is a positive parameter, and [
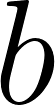
](https://www.codecogs.com/eqnedit.php?latex=b#0) is a parameter with values between 2/3 and 1. Here, we assumed that the number of arthropods that the resin can trap is proportional to either the volume of the resin or its surface area. Thus, these values of [
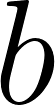
](https://www.codecogs.com/eqnedit.php?latex=b#0) were chosen because the volume of the resin is proportional to its mass, and the surface area is therefore proportional to [
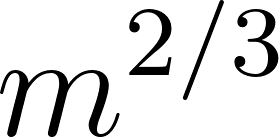
](https://www.codecogs.com/eqnedit.php?latex=m%5E%7B2%2F3%7D#0). If the resin can trap arthropods throughout its entire volume, then [
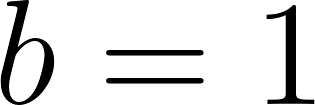
](https://www.codecogs.com/eqnedit.php?latex=b%20%3D%201#0); if it can only trap at the surface, then [
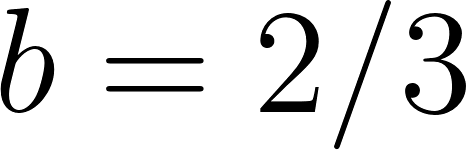
](https://www.codecogs.com/eqnedit.php?latex=b%20%3D%202%2F3#0). The fit resulted in similar exponents: [
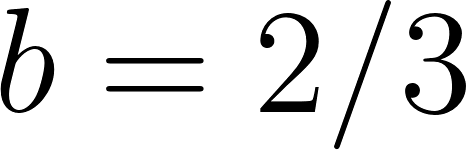
](https://www.codecogs.com/eqnedit.php?latex=b%20%3D%202%2F3#0) for *Hymenaea* and [
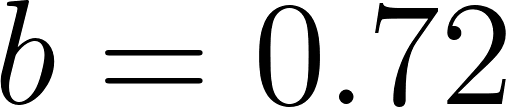
](https://www.codecogs.com/eqnedit.php?latex=b%20%3D%200.72#0) for *Agathis*. Removing the [
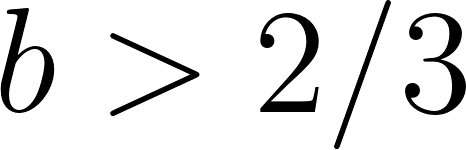
](https://www.codecogs.com/eqnedit.php?latex=b%20%3E%202%2F3#0) restriction results in the same exponent for *Agathis*, but for *Hymenaea*, the exponent becomes [
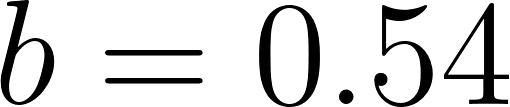
](https://www.codecogs.com/eqnedit.php?latex=b%20%3D%200.54#0). We attribute this discrepancy to the minimal value of [
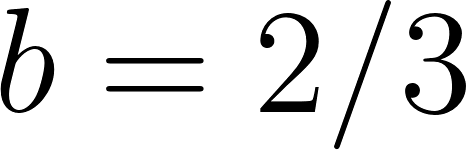
](https://www.codecogs.com/eqnedit.php?latex=b%20%3D%202%2F3#0) and the dispersion of the data.

**Uniformity analysis.** The quality of a trap depends on two factors: the number of arthropods it can capture and the degree of bias (in terms of ecosystem sampling effectiveness) of the trap. A good trap should not only capture a high density of arthropods, but it should also do so with minimal bias, which means it should be more uniform. For example, in Fig. S1, we present three different scenarios for resin inclusions (points): hyperuniform (A), uniform (B), and non-uniform (C). All the figures have the same number of inclusions; they only differ in their distribution. Scenario (A) is unlikely because it requires a strong spatial correlation between inclusions, which does not occur. However, scenarios (B) and (C) are quite possible. If the inclusions are distributed uniformly as in (B), this means that there is less bias in the resin, at least in the position, i.e., it is equally probable to catch an arthropod in any place where the resin is produced in the forest. Therefore, measuring the uniformity of inclusion distribution helps categorize resin quality as a trap and its ecosystem sampling effectiveness; greater uniformity indicates higher quality.

The data in Table 1 and the representation in Fig. 2 suggest that *Agathis* resin is less efficient at trapping arthropods than *Hymenaea* resin (especially for the *Hymenaea* *verrucosa* resin from Madagascar) as it achieves a lower density of inclusions than *Hymenaea* trees, However, these results do not provide information on the effectiveness of the resin as a trap.

To measure the degree of uniformity, we examine the distribution of resin pieces without arthropod inclusions from each collection, *i.e*., New Caledonia (*Agathis lanceolata* and *A. ovata*) and Madagascar (*Hymenaea verrucosa*) (see Additional file 4). If the arthropod inclusions are uniformly distributed in the resin, it implies they follow a Poisson process. Equivalently, since the density is constant and therefore the mass m is proportional to the volume. Therefore, the probability density function (PDF) P(m) of lacking arthropod inclusions, given that the piece has a mass m follows an exponential function of the form $P\left( m \right) = e^{-am}$, where ‘a’ is a constant parameter depending on the density of arthropods [92]. A non-uniform distribution, on the other hand, would deviate from the exponential distribution to a distribution that decreases more slowly. Therefore, for large enough m, we can expect that a power law will fit the PDF, i.e.,

$$P\left( m \right) = P\left( m \right)= am^{-b},$$

where ‘a’ is the value of the PDF for one gram of resin, and ‘b’ is a positive number that measures the degree of non-uniformity in the distribution (the larger it is, the more uniform the distribution).

Note that the probability density function of no inclusions given a mass m, P(m) = P(No inclusions|m), is not the same as the probability density function of m given that there are no inclusions, i.e., P(m|No inclusions). The latter can be estimated by counting the number of pieces of mass m with no inclusions, and dividing it by the total number of pieces without inclusions. To estimate P(m), we can use P(m|No inclusions) and Bayes’ theorem:

$$P\left( B \right) = \frac{ƩA P\left( A \right)P\left( A \right)}{P\left( B \right)}$$

In this case, A is equivalent to ‘No inclusions’, and B is equivalent to m. Thus, P(A) can be estimated by the ratio of the number of pieces without inclusions to the total number of pieces; P(B) can be estimated by the ratio of the number of pieces with a specific volume to the total number of pieces. Once we have P(m), we fit P(m) using the least squares algorithm with the power law to obtain ‘b’, which serves as a measure of the uniformity of the inclusions.


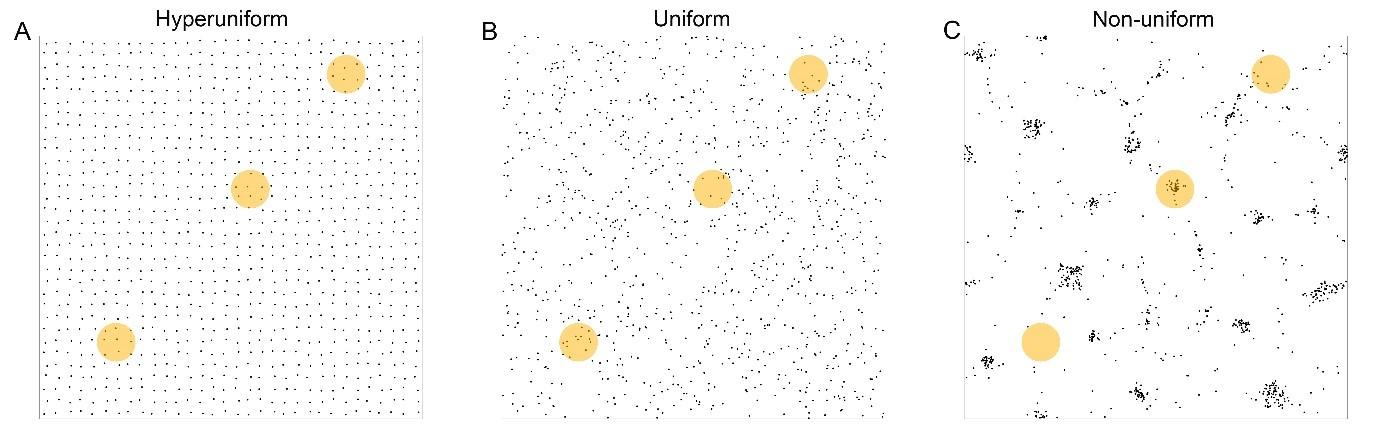


**Fig. S1.** Theoretical distributions of bioinclusions in a piece of resin. The distribution of bioinclusions was calculated with 1,000 points. **A**, represents the hyperuniform distribution, which is not the case of a natural distribution in resin. **B**, represents the uniform distribution. **C**, represents the non-uniform distribution.

**Fig. S2.** Diptera families entrapped around and close to *Agathis* *lanceolata* by yellow sticky traps at 0, 1, and 2 meters high, and by Malaise traps, respectively. Abbreviations: **T ST**: Total yellow sticky traps at 0, 1, and 2 meters; **M**: Malaise trap.


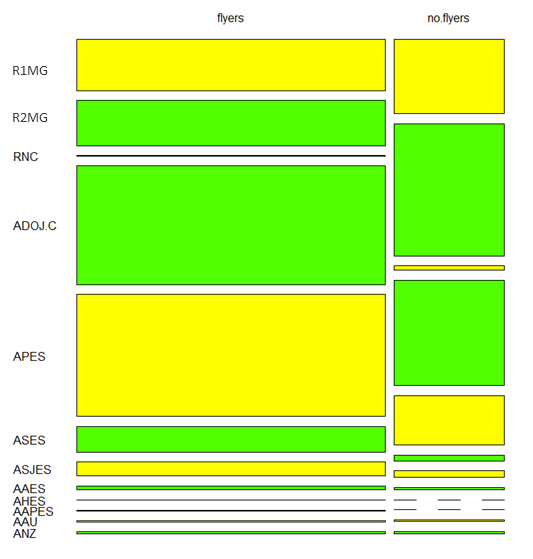


**Fig. S3.** Mosaic plot analysis to represent the results of the observed relative frequencies between deposits and flying and non-flying insects. This mosaic plot shows the relationship between insect groups (flyers and non-flyers) and the deposits in which they occur. The horizontal axis represents the two insect groups, with column widths proportional to the number of observations in each group. The vertical axis within each column represents the deposits, with segment heights proportional to the number of observations within each deposit for that particular insect group. This visualisation allows for the identification of patterns and comparisons between the distribution of deposits across the flyers and non-flyers insect groups, revealing potential associations. **Abbreviations**: **AAES**: Amber from Ariño, Spain; **AAPES**: Amber from Arroyo de la Pascueta, Spain; **ADOJ.C**: Dominican amber from the private collection J. Caridad; **AHES**: Amber from La Hoya, Spain; **APES**: Amber form Peñacerrada, Spain; **ASES**: Amber from El Soplao, Spain; **ASJES**: Amber from San Just, Spain; **AAU**: Amber from Anglesea Coal Measures, Australia; **ANZ**: Amber from Southland region of the South Island, and Otago, New Zealand; **R1MG**: Resin collected in *Hymenaea* in 2013 in Madagascar; **R2MG**: Resin collected in *Hymenaea* in 2015 in Madagascar; **RNC**: Resin collected in *Agathis ovata* and *A. lanceolata* in 2016 in New Caledonia.


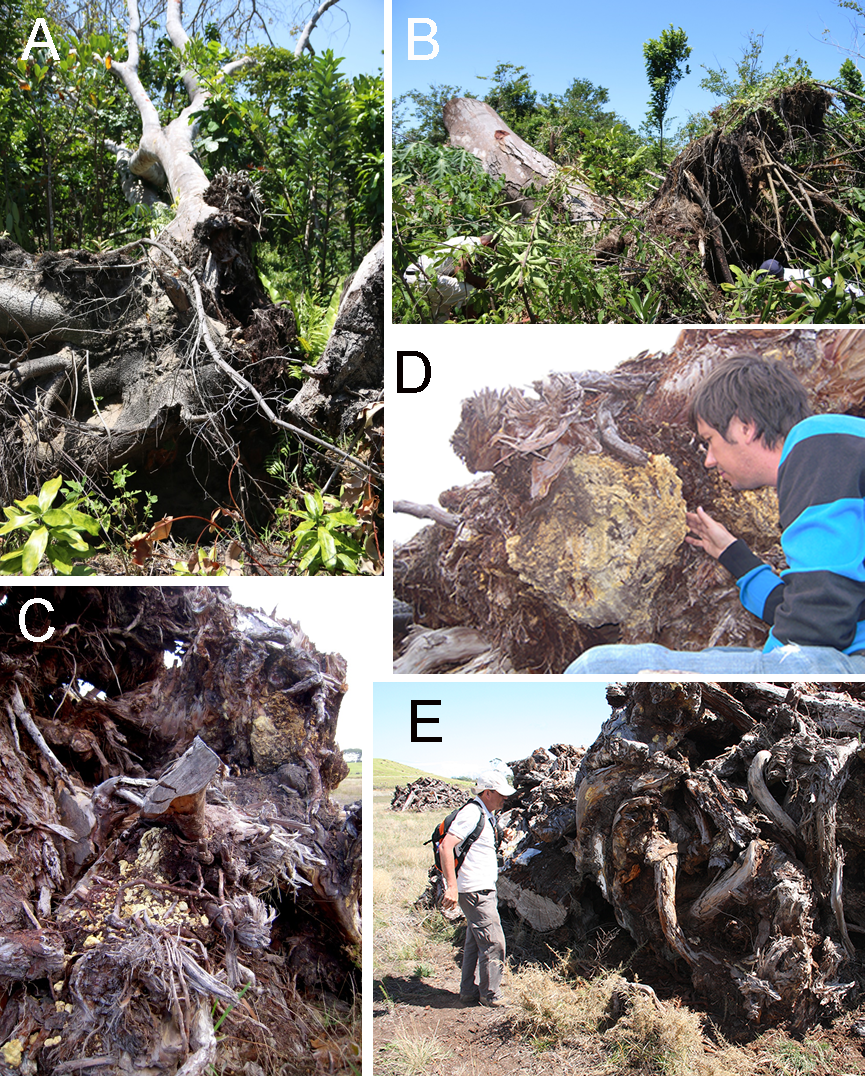


**Fig. S4.** Roots of resin-producing trees. **A** and **B**. Roots of *Hymenaea verrucosa* trees on the east coast, close to Sambava, Madagascar, after the hurricane period in April 2017. **C**, ,**D** and **E**, Pleistocene *Agathis australis* roots abandoned on a private property in Waipapakauri (northern New Zealand, 2011) with large clumps of Pleistocene copal. The ancient trees were dug up, but only the trunks were used for manufacturing.

**
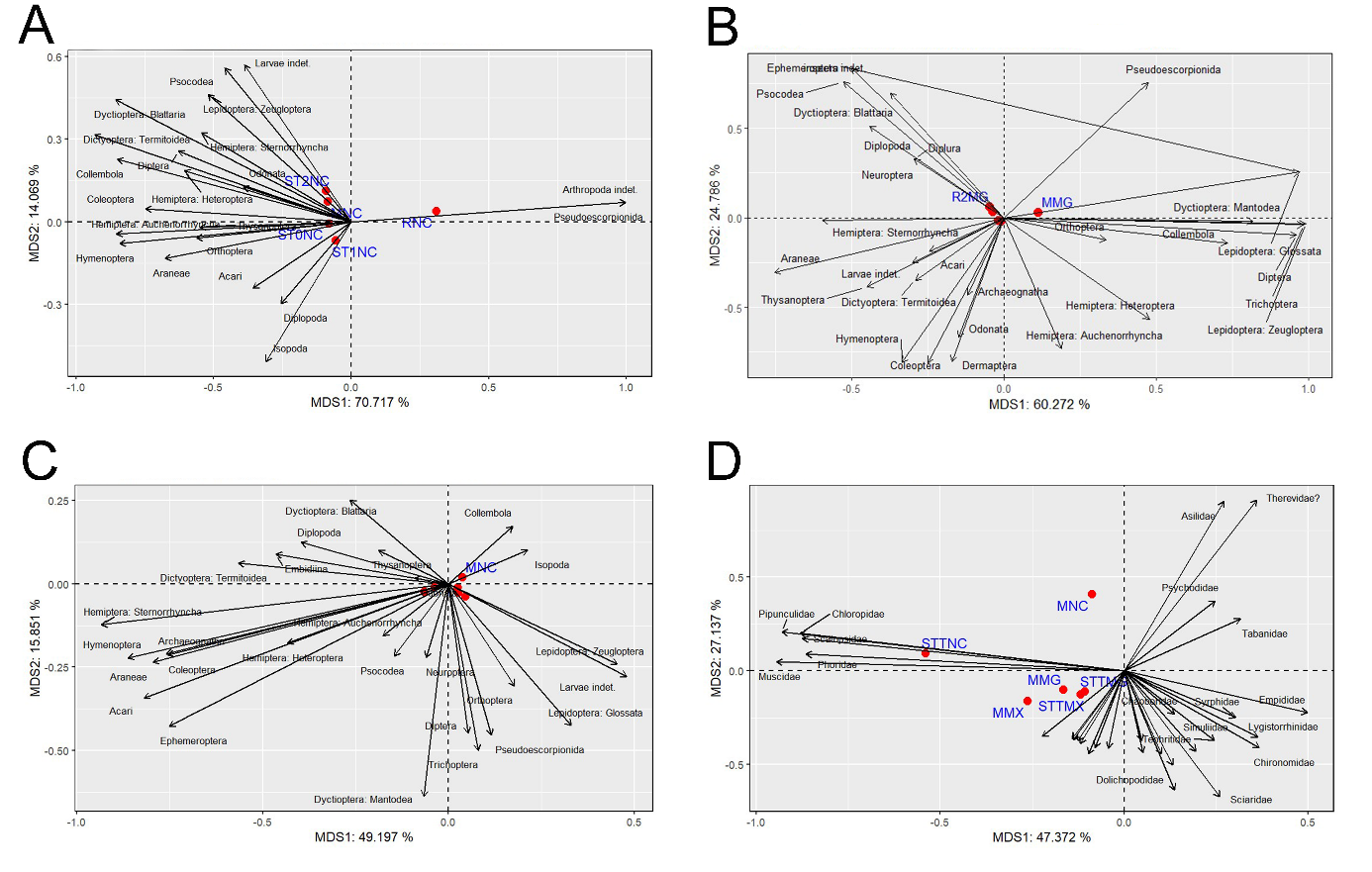
**

**Fig. S5.** Multidimensional scaling (MDS) showing the vectors of influence using Bhattacharyya distance for arthropod orders (A-C) and Diptera families (D) trapped on the trunk and close to *Agathis* and *Hymenaea* trees. **A** by the yellow sticky and Malaise traps and by Defaunation resin in an *Agathis* forests in New Caledonia. **B** by the yellow sticky and Malaise traps and by Defaunation resin in a *Hymeaea* forests in Madagascar. **C** by the yellow sticky and Malaise traps in an *Agathis* forest in New Caledonia and in an *Hymenaea* forests in Madagascar and Mexico. **D** by yellow sticky and Malaise traps in an *Agathis* forest in New Caledonia and in an *Hymenaea* forests in Madagascar and Mexico. The MDS plot shows the proportion of variance explained by each component of the Bhattacharyya distance scaling for each axis. The prefixes ST and M stand for yellow sticky and Malaise traps respectively, and R for resin (R1 collected in 2013, and R2 collected in 2015). ST followed by a number represents the height in metres at which the yellow sticky trap was placed on the tree trunk. T represents the sum of arthropods trapped in sticky traps in one location. The suffixes NC, MG, and MX stand for New Caledonia, Madagascar, and Mexico respectively. A biplot is used to simultaneously visualize the samples and the taxa that influence their position.

**Correlation of variables for the MDS biplot Fig. S5A**

| **Variable** | **MDS 1** | **MDS 2** |
| --- | --- | --- |
| Acari | -0.3568302 | -0.24130144 |
| Amphipoda indet. |  |  |
| Arachnida indet. |  |  |
| Araneae | -0.6770995 | -0.13344609 |
| Archaeognatha |  |  |
| Chilopoda |  |  |
| Coleoptera | -0.7478859 | 0.04758537 |
| Collembola | -0.8508001 | 0.22651626 |
| Dermaptera |  |  |
| Diplopoda | -0.2550289 | -0.29678092 |
| Diplostraca: Cladocera |  |  |
| Diplura |  |  |
| Diptera | -0.6282650 | 0.25717715 |
| Dyctioptera: Blattaria | -0.8567088 | 0.44400322 |
| Dyctioptera: Mantodea |  |  |
| Dictyoptera: Termitoidea | -0.9330359 | 0.31608507 |
| Dyctioptera indet. |  |  |
| Embidiina |  |  |
| Ephemeroptera |  |  |
| Hemiptera |  |  |
| Hemiptera: Auchenorrhyncha | -0.8535970 | -0.04391620 |
| Hemiptera: Heteroptera | -0.6060185 | 0.18522253 |
| Hemiptera: Sternorrhyncha | -0.5448327 | 0.32243140 |
| Hymenoptera | -0.8420503 | -0.07893045 |
| Isopoda | -0.3102526 | -0.50755941 |
| Lepidoptera: Glossata |  |  |
| Lepidoptera: Zeugloptera | -0.5206987 | 0.46211986 |
| Lepidoptera Larvae |  |  |
| Lophioneurida |  |  |
| Mecoptera |  |  |
| Neuroptera |  |  |
| Odonata | -0.3926061 | 0.12715744 |
| Opiliones |  |  |
| Orthoptera | -0.5623023 | -0.05876146 |
| Podocopa? |  |  |
| Pseudoescorpionida | 0.9972469 | 0.06993058 |
| Psocodea | -0.4593039 | 0.55817135 |
| Raphidioptera |  |  |
| Scorpiones |  |  |
| Strepsiptera |  |  |
| Tanaidacea |  |  |
| Thysanoptera | -0.5494980 | -0.01992202 |
| Trichoptera |  |  |
| Zoraptera |  |  |
| Zygentoma |  |  |
| Larvae indet. | -0.3888157 | 0.57027297 |
| insects indet. |  |  |
| Arthropoda indet. | 0.9972469 | 0.06993058 |

**Correlation of variables for the MDS biplot Fig. S5B**

| **Variable** | **MDS 1** | **MDS 2** |
| --- | --- | --- |
| Acari | -0.2426220 | -0.18748562 |
| Amphipoda indet. |  |  |
| Arachnida indet. |  |  |
| Araneae | -0.7519181 | -0.30399164 |
| Archaeognatha | -0.1182069 | -0.43136415 |
| Chilopoda |  |  |
| Coleoptera | -0.2493009 | -0.81026569 |
| Collembola | 0.7346047 | -0.14025974 |
| Dermaptera | -0.1696917 | -0.80277830 |
| Diplopoda | -0.2938228 | 0.32651819 |
| Diplostraca: Cladocera |  |  |
| Diplura | -0.2938228 | 0.32651819 |
| Diptera | 0.9609899 | -0.09900628 |
| Dyctioptera: Blattaria | -0.4406020 | 0.51060448 |
| Dyctioptera: Mantodea | 0.8172379 | -0.01787063 |
| Dictyoptera: Termitoidea | -0.2896568 | -0.34849087 |
| Dyctioptera indet. |  |  |
| Embidiina | -0.3710832 | 0.69588784 |
| Ephemeroptera | 0.9707468 | 0.25490171 |
| Hemiptera |  |  |
| Hemiptera: Auchenorrhyncha | 0.1890366 | -0.73096831 |
| Hemiptera: Heteroptera | 0.4782827 | -0.56692622 |
| Hemiptera: Sternorrhyncha | -0.5961373 | -0.01782573 |
| Hymenoptera | -0.3335602 | -0.80978266 |
| Isopoda | -0.3710832 | 0.69588784 |
| Lepidoptera: Glossata | 0.9707468 | 0.25490171 |
| Lepidoptera: Zeugloptera | 0.9914913 | -0.03466345 |
| Lepidoptera Larvae | -0.3710832 | 0.69588784 |
| Lophioneurida |  |  |
| Mecoptera |  |  |
| Neuroptera | -0.2938228 | 0.32651819 |
| Odonata | -0.1483377 | -0.66877714 |
| Opiliones |  |  |
| Orthoptera | 0.3351126 | -0.12506020 |
| Podocopa? |  |  |
| Pseudoescorpionida | 0.4740757 | 0.75166514 |
| Psocodea | -0.5254563 | 0.75786309 |
| Raphidioptera |  |  |
| Scorpiones |  |  |
| Strepsiptera |  |  |
| Tanaidacea |  |  |
| Thysanoptera | -0.4484410 | -0.38740553 |
| Trichoptera | 0.9838545 | -0.04030598 |
| Zoraptera |  |  |
| Zygentoma | -0.3710832 | 0.69588784 |
| Larvae indet. | -0.2998917 | -0.24923253 |
| insects indet. | -0.4995070 | 0.83472108 |
| Arthropoda indet. | -0.3710832 | 0.69588784 |

**Correlation of variables for the MDS biplot Fig. S5C.**

| **Variable** | **MDS 1** | **MDS 2** |
| --- | --- | --- |
| Acari | -0.81946414 | -0.34191241 |
| Amphipoda indet. |  |  |
| Arachnida indet. |  |  |
| Araneae | -0.79410904 | -0.23489003 |
| Archaeognatha | -0.75974316 | -0.21062966 |
| Chilopoda |  |  |
| Coleoptera | -0.75472851 | -0.21336593 |
| Collembola | 0.17217560 | 0.17320438 |
| Dermaptera |  |  |
| Diplopoda | -0.39702330 | 0.12427142 |
| Diplostraca: Cladocera |  |  |
| Diplura |  |  |
| Diptera | 0.05170745 | -0.44838409 |
| Dyctioptera: Blattaria | -0.26490397 | 0.25299220 |
| Dyctioptera: Mantodea | -0.06663374 | -0.63822518 |
| Dictyoptera: Termitoidea | -0.56510506 | 0.06167121 |
| Dyctioptera indet. |  |  |
| Embidiina | -0.46362911 | 0.08900841 |
| Ephemeroptera | -0.75120977 | -0.42752909 |
| Hemiptera |  |  |
| Hemiptera: Auchenorrhyncha | -0.17639671 | -0.15612766 |
| Hemiptera: Heteroptera | -0.43347926 | -0.17866901 |
| Hemiptera: Sternorrhyncha | -0.93392249 | -0.12279366 |
| Hymenoptera | -0.86226417 | -0.22222058 |
| Isopoda | 0.21312590 | 0.10162339 |
| Lepidoptera: Glossata | 0.32993425 | -0.42587433 |
| Lepidoptera: Zeugloptera | 0.45392495 | -0.24106675 |
| Lepidoptera Larvae |  |  |
| Lophioneurida |  |  |
| Mecoptera |  |  |
| Neuroptera | -0.06075828 | -0.22144544 |
| Odonata | -0.09077136 | 0.01737153 |
| Opiliones |  |  |
| Orthoptera | 0.17760006 | -0.30632582 |
| Podocopa? |  |  |
| Pseudoescorpionida | 0.11526768 | -0.45415898 |
| Psocodea | -0.14601633 | -0.21703272 |
| Raphidioptera |  |  |
| Scorpiones |  |  |
| Strepsiptera |  |  |
| Tanaidacea |  |  |
| Thysanoptera | -0.18823278 | 0.09943485 |
| Trichoptera | 0.08015724 | -0.49984857 |
| Zoraptera |  |  |
| Zygentoma |  |  |
| Larvae indet. | 0.47949149 | -0.27815645 |
| insects indet. |  |  |
| Arthropoda indet. |  |  |

**Correlation of variables for the MDS biplot Fig. S5D**

| **Variable** | **MDS 1** | **MDS 2** |
| --- | --- | --- |
| Anisopodidae | -0.14212228 | -0.36328003 |
| †Archizelmiridae |  |  |
| Atelestidae |  |  |
| Asilidae | 0.27122511 | 0.90459802 |
| Bibionidae |  |  |
| Bombilidae |  |  |
| Cecidomyiidae | -0.11931038 | -0.38745373 |
| Ceratopogonidae | 0.10110260 | -0.44283868 |
| Chaoboridae | 0.13585210 | -0.23021876 |
| Chimeromyiidae |  |  |
| Chironomidae | 0.36741517 | -0.40956590 |
| Chloropidae | -0.88216576 | 0.20082465 |
| Corethrelidae | -0.09734481 | -0.44292527 |
| Culicidae | -0.04276993 | -0.41252601 |
| Dixidae |  |  |
| Dolichopodidae | 0.13737155 | -0.63546129 |
| Drosophilidae | -0.07949793 | -0.40867018 |
| Empididae | 0.49798807 | -0.22303609 |
| Hybotidae |  |  |
| Ironomyiidae |  |  |
| Keroplatidae | 0.04698086 | -0.37220120 |
| Limnophilidae |  |  |
| Limoniidae | -0.13964235 | -0.36785045 |
| Lonchopteridae |  |  |
| Lygistorrhinidae | 0.36306745 | -0.35208696 |
| Micropharidae? |  |  |
| Milichidae |  |  |
| Micropezidae | 0.13171358 | -0.50296514 |
| Muscidae | -0.94541315 | 0.04720403 |
| Mycetophilidae | -0.12665782 | -0.37068115 |
| Neriidae |  |  |
| Phoridae | -0.86565935 | 0.08859386 |
| Platypecidae |  |  |
| Pipunculidae | -0.93037237 | 0.20804105 |
| Psychodidae | 0.24711650 | 0.37398948 |
| Ptychopteridae |  |  |
| Rhagionidae |  |  |
| Scatopsidae | -0.87441843 | 0.17140239 |
| Sciaridae | 0.25978707 | -0.67067621 |
| Scenopinidae? |  |  |
| Simuliidae | 0.30323569 | -0.24873749 |
| Stratiomyomorpha | -0.22261017 | -0.35060018 |
| Syrphidae | 0.30323569 | -0.24873749 |
| Tabanidae | 0.31606507 | 0.27767224 |
| Tachinidae |  |  |
| Tabanomorpha |  |  |
| Tanyderidae |  |  |
| Tephritidae | 0.24541490 | -0.37221468 |
| †Tethepomyiidae |  |  |
| Therevidae? | 0.35956240 | 0.91212435 |
| Tipulidae | -0.14212228 | -0.363280 |


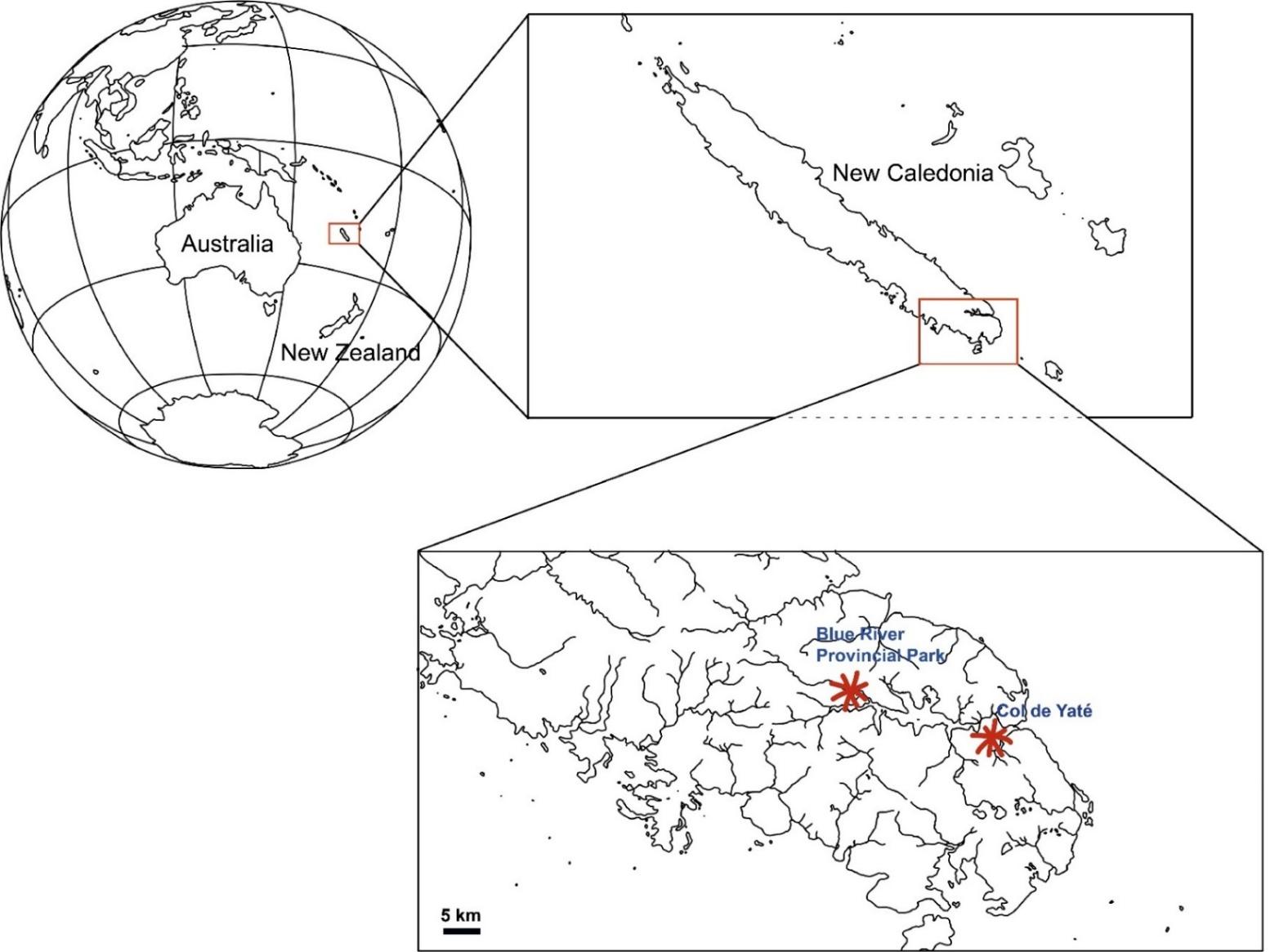


**Fig. S6.** Map showing the localisation of New Caledonia and the sampling localities cited in the text.
